# *Pseudomonas aeruginosa-*mediated cardiac dysfunction is driven by extracellular vesicles released during infection

**DOI:** 10.1128/mbio.03091-25

**Published:** 2026-01-15

**Authors:** Naresh Kumar, Sameer Salam Matoo, Shridhar Sanghvi, Maneeth P. Ellendula, Sahil Mahajan, Clara Planner, Joseph S. Bednash, Mahmood Khan, Latha P. Ganesan, Harpreet Singh, William P. Lafuse, Daniel J. Wozniak, Murugesan V. S. Rajaram

**Affiliations:** 1 Department of Microbial Infection and Immunity, The Ohio State University 2647, Columbus, Ohio, USA; 2 Department of Physiology and Cell Biology, The Ohio State University 2647, Columbus, Ohio, USA; 3 Department of Internal Medicine, The Ohio State University 2647, Columbus, Ohio, USA; 4 Division of Basic and Translational Research, Department of Emergency Medicine, The Ohio State University 2647, Columbus, Ohio, USA; 5 Department of Microbiology, The Ohio State University 2647, Columbus, Ohio, USA; Massachusetts General Hospital, Boston, Massachusetts, USA

**Keywords:** *Pseudomonas aeruginosa*, cardiac dysfunction, microelectrode array (MEA), outer membrane vesicles (OMVs), exosomes, human induced pluripotent stem cell-derived cardiomyocytes (hiPSC-CMs)

## Abstract

*Pseudomonas aeruginosa* is a gram-negative, opportunistic pathogen and a major cause of severe pneumonia. Healthcare-associated pneumonia accounts for up to 22% of all healthcare-acquired infections, with *P. aeruginosa* contributing to approximately 10–20% of these cases. Infections caused by *P. aeruginosa* carry a high mortality rate, ranging from 32% to 42.8%. Notably, the risk of pneumonia is strongly associated with cardiovascular diseases (CVD), particularly heart failure, independent of age, sex, comorbidities, or antibiotic usage. Individuals with CVD are at increased risk of developing both hospital-acquired pneumonia (HAP) and community-acquired pneumonia (CAP). Despite this association, the mechanisms underlying infection-induced cardiac dysfunction remain poorly understood. In our previous studies, we demonstrated that *P. aeruginosa* lung infection leads to severe cardiac electrical disturbances, including arrhythmias and left ventricular (LV) dysfunction, despite minimal bacterial dissemination to the heart. To further elucidate the mechanisms of *P. aeruginosa*-induced cardiac dysfunction, we employed both *in vitro* and *in vivo* infection models. Exposure of human-induced pluripotent stem cell-derived cardiomyocytes (hiPSC-CMs) to conditioned media from *P. aeruginosa*-infected human macrophages (hMDMs) resulted in profound contractile dysfunction in the hiPSC-CMs. We identified vesicles released from infected hMDMs along with bacterial outer membrane vesicles (OMVs) as key mediators of this dysfunction. Using liquid chromatography-tandem mass spectrometry (LC-MS/MS), we identified bacterial proteins, including toxins, packaged within both exosomes and OMVs that were responsible for the cardiotoxic effects. Moreover, systemic administration of bacterial OMVs in mice led to severe cardiac dysfunction. In summary, our findings indicate that during *P. aeruginosa* infection, bacterial OMVs are released into circulation and play a central role in mediating cardiac dysfunction.

**IMPORTANCE:**

Bacterial pneumonia can lead to severe cardiovascular complications and is a major contributor to increased mortality among hospitalized patients, either directly or indirectly. *Pseudomonas aeruginosa*, an opportunistic pathogen frequently encountered in hospital settings, accounts for nearly 20% of all infections in intensive care units (ICUs). Our previous studies demonstrated that *P. aeruginosa* lung infection induces profound cardiac electrical abnormalities and left ventricular (LV) dysfunction, despite minimal bacterial dissemination to the heart. In the present study, we identify exosomes released from infected host cells and outer membrane vesicles (OMVs) secreted by *P. aeruginosa* as critical mediators of this cardiac dysfunction. We show that host-derived exosomes are enriched with bacterial OMVs containing toxins and other immunogenic molecules, which promote systemic inflammation and tissue injury, ultimately contributing to cardiac impairment.

## INTRODUCTION

In the United States, over 5 million people develop pneumonia annually, making it the 8th leading cause of death (1). *Pseudomonas aeruginosa* (*P.a*.), a gram-negative opportunistic pathogen, is commonly found in both environment and healthcare settings (2, 3). It is responsible for a range of infections, including pneumonia, urinary tract infections, and bloodstream infections (4). Although these *P.a*. infections can affect various parts of the body, lung infections are particularly serious and can lead to life-threatening conditions with higher mortality rates (5). In the healthcare environment, *P.a*. accounts for 10–20% of all ventilated pneumonia, with mortality rates remaining notably high (6). Additionally, up to 30% of patients hospitalized with pneumonia develop cardiovascular complications (7). Conversely, individuals with pre-existing cardiovascular diseases (CVD) experience frequent hospitalizations for cardiac conditions, which increase their risk of acquiring healthcare-associated infections (HAIs), including *P.a*. These infections can further exacerbate cardiac dysfunction or precipitate heart failure. Several studies have reported a strong association between pneumonia and CVD (5, 8–10), and our recent work demonstrated that *P.a*. infection induces cardiac dysfunction despite minimal bacterial dissemination into heart tissue (11). However, the mechanism of CVD during *P.a*. infection remains poorly understood. We hypothesize that effector molecules, such as host-derived exosomes from infected cells and bacterial outer membrane vesicles (OMVs), are released during infection, traveled from the lungs into circulation, and mediate cardiovascular complications.

Once *P.a*. infection is established in the lungs (as in pneumonia) or at other infection sites, the bacteria release bacterial pathogen-associated molecular patterns (PAMPs) and OMVs. In response, infected host cells release inflammatory mediators, such as IL-1β, TNF-α, IL-6, and damage-associated molecular patterns (DAMPs), into the bloodstream, which trigger systemic inflammation and tissue damage. Gram-negative bacteria secrete nanosized OMVs that contain a diverse array of immunogenic molecules, including endotoxins, enzymes, peptidoglycan, periplasmic proteins, short RNAs (sRNAs), and nucleic acids (12–14). These vesicles range from 10 to 300 nm (15–17) in diameter and play a vital role in host-pathogen interactions by facilitating intracellular communication, modulating host immune responses, and mediating effects in distal organs (12–14). Similarly, host cells release extracellular vesicles (EVs), which are categorized based on their size and biogenesis. EVs larger than 100–1,000 nm are called microvesicles, 50–5,000 nm are called apoptotic bodies, and EVs with an average size of 40–160 nm are called exosomes (18–21). Earlier studies demonstrated that the process of exosome secretion is a way to release cellular waste (18, 22, 23). However, subsequent studies revealed that exosomes carry important proteins, lipids, and genetic materials that are essential for intercellular communication. Exosome-mediated signaling plays an important role in both physiological and pathological processes, including cardiovascular health. In particular, exosomes have been implicated in the progression of cardiovascular complications such as heart failure, myocardial ischemia, and atherosclerosis (24–26).

The human-induced pluripotent stem cell-derived cardiomyocytes (hiPSC-CMs) have emerged as a robust *in vitro* model to study the mechanisms underlying cardiomyopathies, cardiotoxicity, and to perform drug screening (27–29). Advances in hiPSC generation, the scalability of hiPSC-CMs production, and the development of next-generation genetic manipulation methods have further enhanced their value as a platform for patient-specific precision medicine (29, 30). Furthermore, hiPSC-CMs express similar cardiac ion channels as mature adult cardiomyocytes and display action potential (AP) durations similar to the human QT interval (31, 32). Therefore, in this study, we utilized hiPSC-CMs as a model system to assess cardiomyocyte contractile function during bacterial infection and dysfunction induced by bacterial-derived components.

In the current study, we treated hiPSC-CMs with conditioned media (C-media) harvested from *P.a*.-infected human monocyte-derived macrophages (hMDMs), or with purified OMVs from *P.a.*, to evaluate cardiomyocyte contractile function. Exposure of hiPSC-CMs to C-media from infected hMDMs caused severe contractile dysfunction, characterized by decreased and irregular beat periods, shortened field potential duration (FPD), and slowed conduction velocity. Furthermore, C-media exposure induced arrhythmogenic activity, as evidenced by prolonged APs with early afterdepolarization (EAD) shoulders, increased action potential durations (APDs), triangulation ratio, and rise time. Similarly, exposure of hiPCS-CMS to purified bacterial OMVs reproduced these contractile and electrical abnormalities. To validate these findings *in vivo*, we administered *P.a*. systemically in mice and monitored survival and cardiac function. Consistent with our *in vitro* results, OMV-treated mice exhibited severe cardiac electrical disturbance and arrhythmias. In conclusion, our findings suggest that exosomes released from infected host cells and OMVs released from *P.a*. carry both host- and bacterial-derived components that can enter circulation, reach the heart, and contribute to infection-induced cardiac dysfunction.

## RESULTS

### Conditioned media from *P.a*.-infected hMDMs induce cardiomyocyte contractile dysfunction

**Fig 1:**
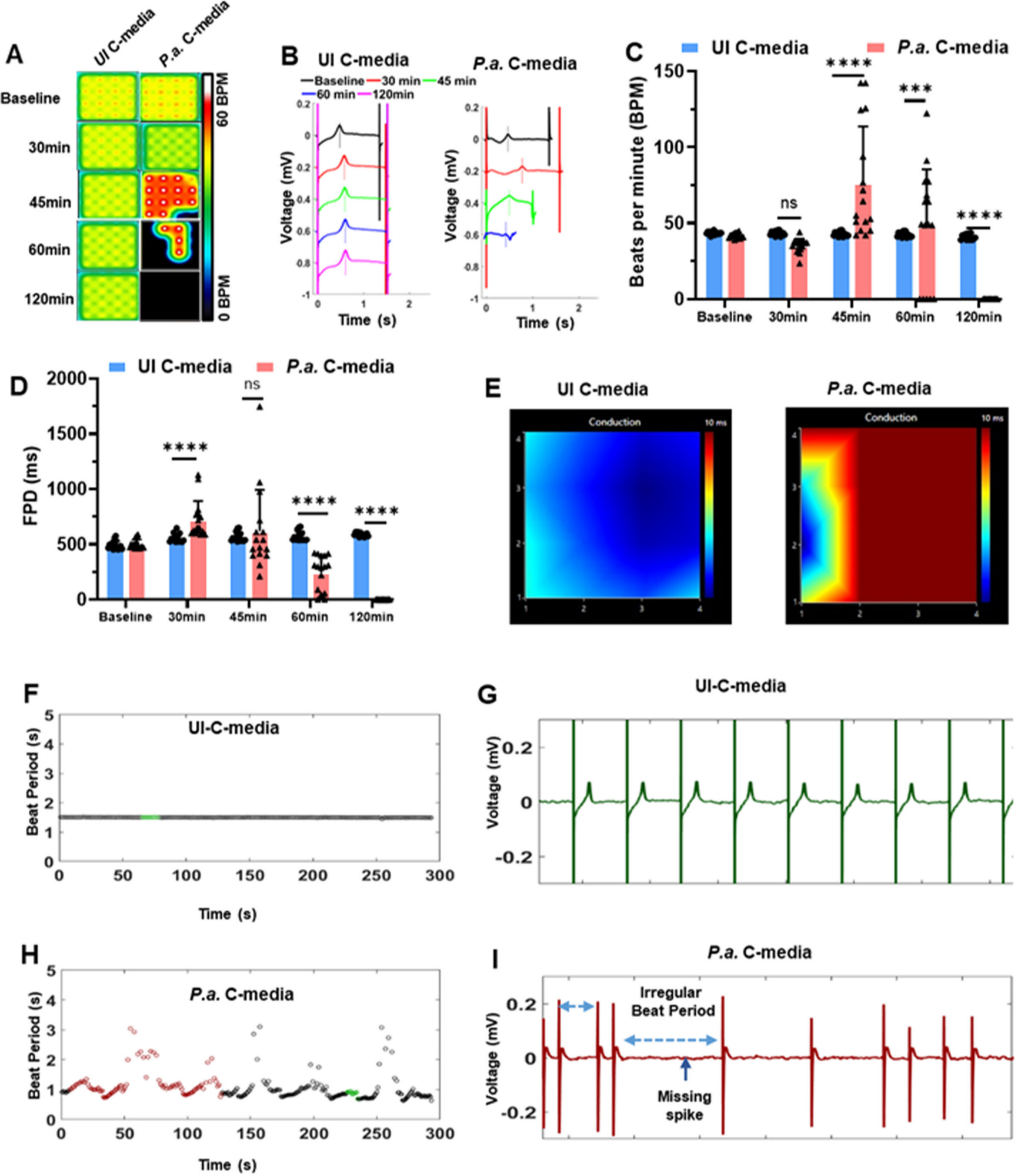
C-media from *P.a*.-infected hMDMs cause cardiomyocyte contractile dysfunction. hiPSC-CMs were plated in 24-well MEA plates, and the cells were exposed to the mixture of cardiomyocyte culture medium and C-media (1:1 ratio) harvested from uninfected (UI C-media) or *P.a*.-infected (*P.a*. C-media) hMDMs. The cardiomyocyte contractility and electrical activity were recorded using AxIS Navigator on the MEA system at 5% CO_2_ and at 37°C for the indicated time points. Data analysis was performed using the cardiac analysis tool. (**A**) Electrical activity map showing the changes in the beat rate of cardiomyocytes treated with UI C-media and *P.a*. C-media. The activity map shown is a representative well from quadruplicate samples for each treatment and four repeats (*N* = 4). (**B**) Representative traces recorded with the MEA system showing the beat period, T-wave, and FPD in UI-C-media- and *P.a*. C-media-treated hiPSC-CMs. (**C**) Beats per minute and (**D**) field potential duration (FPD) at baseline, 30, 45, 60, and 120 min post-treatment of hiPSC-CMs with UI C-media and *P.a*. C-media. (**E**) Representative wave propagation of hiPSC-CMs treated with UI C-media and *P.a*. C-media. (**F–G**) Representative EKG traces of hiPSC-CMs treated with UI C-media (**H–I**) *P.a*. C-media. Data shown in panels **C** and **D** are accumulative data from four independent experiments, mean ± SD: *P < 0.05, **P < 0.01, ***P < 0.001, ****P < 0.0001.

*P.a*. causes lung infections that can lead to severe pneumonia and heart failure (11, 33). We previously demonstrated that *P.a*. pulmonary infection in mice induces cardiac electrical and left ventricular (LV) dysfunction without significant bacterial dissemination to the heart (11), suggesting that host-derived inflammatory mediators and/or bacterial products released into circulation contribute to systemic inflammation-mediated cardiac dysfunction. To determine whether *P.a*. infection causes cardiac dysfunction by releasing bacterial products and inflammatory mediators, we infected hMDMs with *P.a*. and collected C-media at 22-h post-infection (h.p.i.). To assess bacterial burden, colony-forming unit (CFU) assays performed on the cell lysates revealed a bacterial load of 3.6 × 10^8^/mL (Fig. S1A). Cell viability was evaluated by measuring lactate dehydrogenase (LDH) release in the C-media, which showed that more than 80% of infected hMDMs were dead at 22 h (Fig. S1B). To ensure the C-media was free of viable bacteria, we filtered C-media on agar plates and confirmed the absence of bacterial growth (Fig. S1C). These bacteria-free C-media samples were used for subsequent *in vitro* experiments, while C-media from uninfected hMDMs served as controls. hiPSC-CMs cultured on a multielectrode array (MEA) plate were exposed to the collected C-media, and their electrical activity was monitored over 24 h using the AxIS Navigator software. The MEA revealed that hiPSC-CMs treated with C-media from *P.a*.-infected hMDMs exhibited markedly reduced electrical activity (Fig. 1A), evidenced by shorter beat periods at 60 min compared to cells treated with control C-media (Fig. 1B). Exposure to infected C-media initially increased the beat rate at 45 min, followed by a progressive decline, with contractions ceasing by 120 min post-treatment (Fig. 1C). Thus, a transient increase in FPD and beats per minute (BPM) may reflect an early physiological response to infection. Despite the electrical dysfunction, the C-media-treated hiPSC-CMs maintained normal morphology at 3 h post-treatment and maintained viability (60%) up to 24 h post-treatment (Fig. S2A and B). To determine the dose dependency of these effects, hiPSC-CMs were exposed to serial dilution of *P.a*. C-media (6.25%, 12.5%, 25%, and 50%). MEA recordings showed that a higher concentration (50%) of infected C-media significantly inhibited electrical activity at 60 min post-exposure, while the severity of dysfunction decreased with further dilution, and beat rates gradually normalized (Fig. S3A and B). Based on these results, 50% diluted *P.a.* C-media was used for all subsequent experiments.

Next, we measured FPD, which represents the time interval between depolarization and repolarization of cardiomyocytes. hiPSC-CMs treated with C-media harvested from *P.a*.-infected hMDMs showed an initial increase in FPD at 30 min, followed by a significant decrease at 60 and 120 min (Fig. 1D). Furthermore, the conduction map shown in Fig. 1E illustrates the synchronized beating of cardiomyocytes. In this map, blue regions indicate faster wave propagation (reflecting efficient cell-to-cell communication), whereas red regions represent slower propagation, suggesting that the treatment with C-media from *P.a*.-infected hMDMs disrupts cardiomyocyte syncytium and delays electrical conduction. In addition, treatment of hiPSC-CMs with *P.a*. C-media promoted arrhythmic behavior, as evidenced by irregular beat intervals and missed depolarization spikes (Fig. 1F through I).

Because irregular beat periods indicate arrhythmogenic potential, we next analyzed AP (34, 35), using the local extracellular action potential (LEAP) mode of the MEA system. Under normal physiological conditions, inward Na^+^ and Ca^2+^ currents are balanced by the outward K^+^ currents to maintain rhythmic depolarization and repolarization. Compared with controls, hiPSC-CMs treated with *P.a.* C-media displayed prolonged APs with EAD shoulders at different time points (Fig.S4), and approximately 80% of the beats exhibited EADs at both the 30 and 45 min post-treatment (Fig. S4B). Together, these findings demonstrate that C-media from *P.a.*-infected hMDMs induce cardiomyocyte contractile dysfunction, as evidenced by decreased beat rate, shortened FPD, abnormal conduction, and irregular beat periods (abnormal automaticity). The prolonged APs and increased frequency of EADs further suggest a proarrhythmic condition induced by infection-derived mediators.

### *P.a*. infection enhances the release of inflammatory cytokines and extracellular vesicles (EVs) from hMDMs

**Fig 2:**
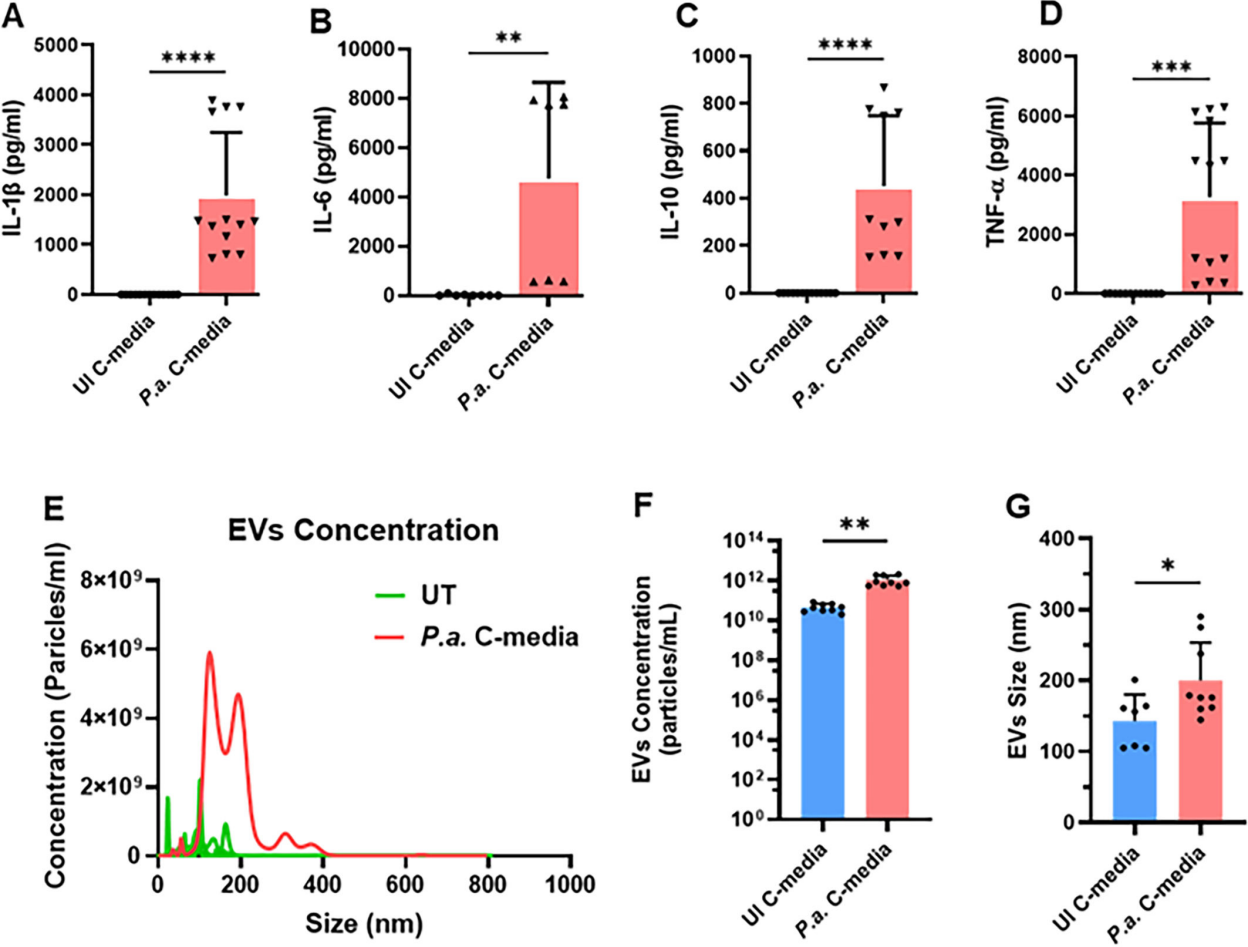
*P*.*a*. infection enhances the release of cytokines and exosomes. hMDMs were infected with *P.a*. (1 MOI) or left uninfected for 22 h. The cell-free culture supernatants were evaluated by ELISA to determine the cytokine production. Graphs shown are (**A**) IL-1β, (**B**) IL-6, (**C**) IL-10, and (**D**) TNF-α levels in C-media. Exosomes isolated from UI-C-media and *P.a*. C-media were quantified using NanoFCM. The plot shown in panel **E** is the distribution of EV size versus their concentration. (**F**) EV concentration and (**G**) EV size. Accumulative data from three independent experiments, mean ± SD: **P < 0.01, ***P < 0.001, ****P < 0.0001.

Infection of hMDMs induces a robust host response, leading to enhanced production of inflammatory cytokines and the release of microvesicles into the surrounding media (36, 37). Therefore, we next examined the levels of inflammatory cytokines and EVs in the C-media collected from *P.a*.-infected hMDMs. Our results showed that *P.a*. infection significantly increased the release of pro-inflammatory cytokines, including IL-1β, IL-6, and TNFα (Fig. 2A through C). Interestingly, we also observed elevated levels of the anti-inflammatory cytokine IL-10 in infected hMDMs compared to uninfected cells (Fig. 2D). Next, we quantified the EVs in the C-media using Nano Analyzer. The analysis revealed that *P.a*. infection markedly enhanced EV release, and the EVs derived from infected hMDMs were larger than those from uninfected cells (Fig. 2E through G). Given the increased levels of both inflammatory cytokines and EVs in C-media from infected hMDMs, we next asked whether heat inactivation of cytokines in the C-media could reduce the contractile dysfunction of hiPSC-CM, using non-heat-inactivated C-media as a control. Surprisingly, our MEA recordings revealed that treatment of hiPSC-CMs with heat-inactivated C-media from *P.a*.-infected hMDMs caused rapid and severe contractile dysfunction compared with non-heat-inactivated C-media (Fig. S5A and B). Quantitative analysis of beat period (Fig. S5C), beat rate, and FPD (Fig. S5D and E) further demonstrated that heat-inactivated C-media led to a more pronounced reduction in beat rate and prolonged depolarization/repolarization, as reflected by increased FPD in this group. These findings suggest that elevated temperature may cause EVs to release their internal contents, including bacterial PAMPs, into the medium, thereby accelerating cardiomyocyte dysfunction.

### Live *P.a*. is required to cause cardiac dysfunction in hiPSC-CMs

**Fig 3:**
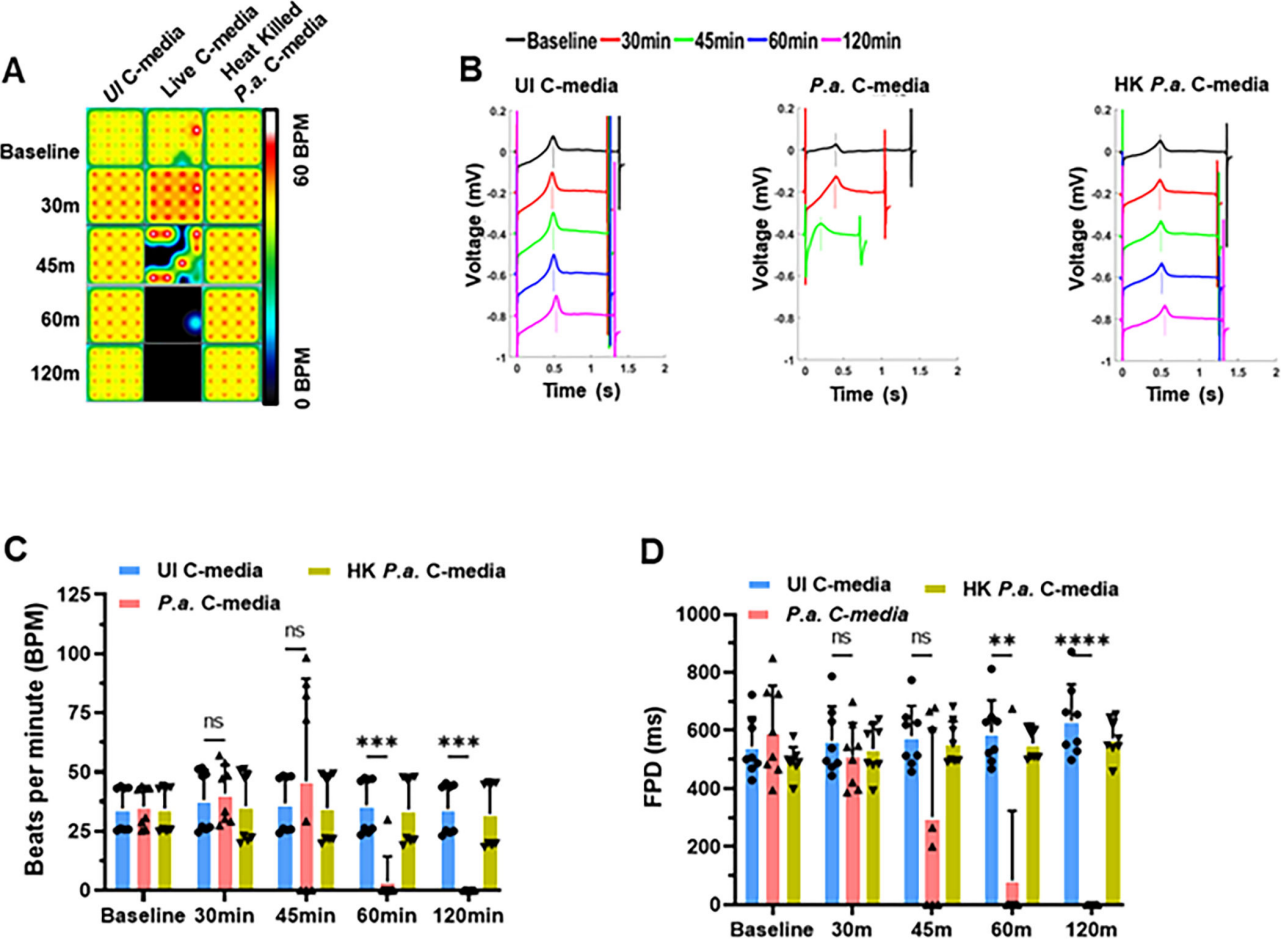
Live bacteria are required to cause cardiomyocyte contractile dysfunction. hiPSC-CMs were plated in 24-well MEA plates, and the cells were exposed to the mixture of cardiomyocyte culture medium and C-media (1:1 ratio) harvested from uninfected (UI C-media), *P.a*.-infected (*P.a*. C-media), or heat-killed *P.a*.-infected (HK *P.a*. C-media) hMDMs. The cardiomyocyte contractility and electrical activity were recorded using AxIS Navigator on the MEA system at 5% CO_2_ and at 37°C for indicated time points. Data analysis was performed using the cardiac analysis tool. (**A**) Electrical activity map showing the changes in beat rate of cardiomyocytes treated with UI C-media and *P.a*. C-media. The activity map shown is a representative well from quadruplicate samples for each treatment and three repeats (*N* = 3). (**B**) Representative traces recorded with the MEA system showing the beat period, T-wave, and FPD in UI-C-media, *P.a*. C-media, and HK *P.a*. C-media-treated hiPSC-CMs. (**C**) Beats per minute and (**D**) field potential duration (FPD) at baseline, 30, 45, 60, and 120 min post-treatment of hiPSC-CMs with UI C-media, *P.a*. C-media, and HK *P.a*. C-media. Data shown in panels **C** and **D** are accumulative data from three independent experiments (mean ± SD; **P < 0.01, ***P < 0.001, ****P < 0.0001). Data shown in panels **E–H** are representative data from two independent experiments, mean ± SD: *P < 0.05, **P < 0.01, ***P < 0.001, ****P < 0.0001.

Next, we investigated whether active bacterial infection is required to induce the production of inflammatory cytokines and EVs in hMDMs. We compared hMDMs infected with live *P.a*. to those exposed to heat-killed bacteria. After 22 h, C-media was harvested and exposed to hiPSC-CMs, and contractile function was monitored using MEA system. These data revealed that live bacterial infection of hMDMs is necessary to induce cardiomyocyte contractile dysfunction (Fig. 3A). Specifically, C-media from live *P.a*.-infected hMDMs caused irregular beat periods, decreased beat rates, and reduced FPD (Fig. 3B through D), whereas C-media from heat-killed bacteria had no effect on cardiomyocyte function. From these studies, two conclusions can be drawn: first, cardiomyocyte contractile dysfunction requires active *P.a*. infection of hMDMs; second, inflammatory cytokines alone in the C-media are insufficient to induce cardiomyocyte contractile dysfunction.

We next assessed whether direct infection of cardiomyocytes with *P.a*. causes more rapid or severe contractile dysfunction. MEA recordings demonstrated that hiPSC-CMs infected directly with live *P.a*. exhibited significant reductions in beat rate and prolonged beat periods by 7 h post-infection (Fig. S6A through C). However, the onset of dysfunction was delayed compared to the rapid effects observed with C-media from infected hMDMs (Fig. 1). The early response to infected C-media likely reflects the immediate availability of bacterial toxins and proteins, which are not present during direct cardiomyocyte infection. Based on these results, we used C-media from *P.a.*-infected hMDMs for all subsequent experiments.

We hypothesized that host-derived molecules from infected cells and/or bacterial products released by live bacteria contribute to cardiomyocyte damage. To test this, hiPSC-CMs were exposed to C-media from *P.a.*-infected hMDMs or from *P.a.* cultured in RPMI medium supplemented with 10% serum, and cardiomyocyte electrical activity was monitored using MEA. The data revealed that C-media from *P.a.* alone induced contractile dysfunction in hiPSC-CMs, as evidenced by altered electrical activity and reduced beat rate (Fig. S7A and B), to a similar extent to C-media from infected hMDMs. These findings suggest that bacterial products released directly from *P.a.* are primarily responsible for inducing cardiomyocyte contractile dysfunction.

### OMVs released from *P.a.* activate cardiomyocyte contractile dysfunction

**Fig 4:**
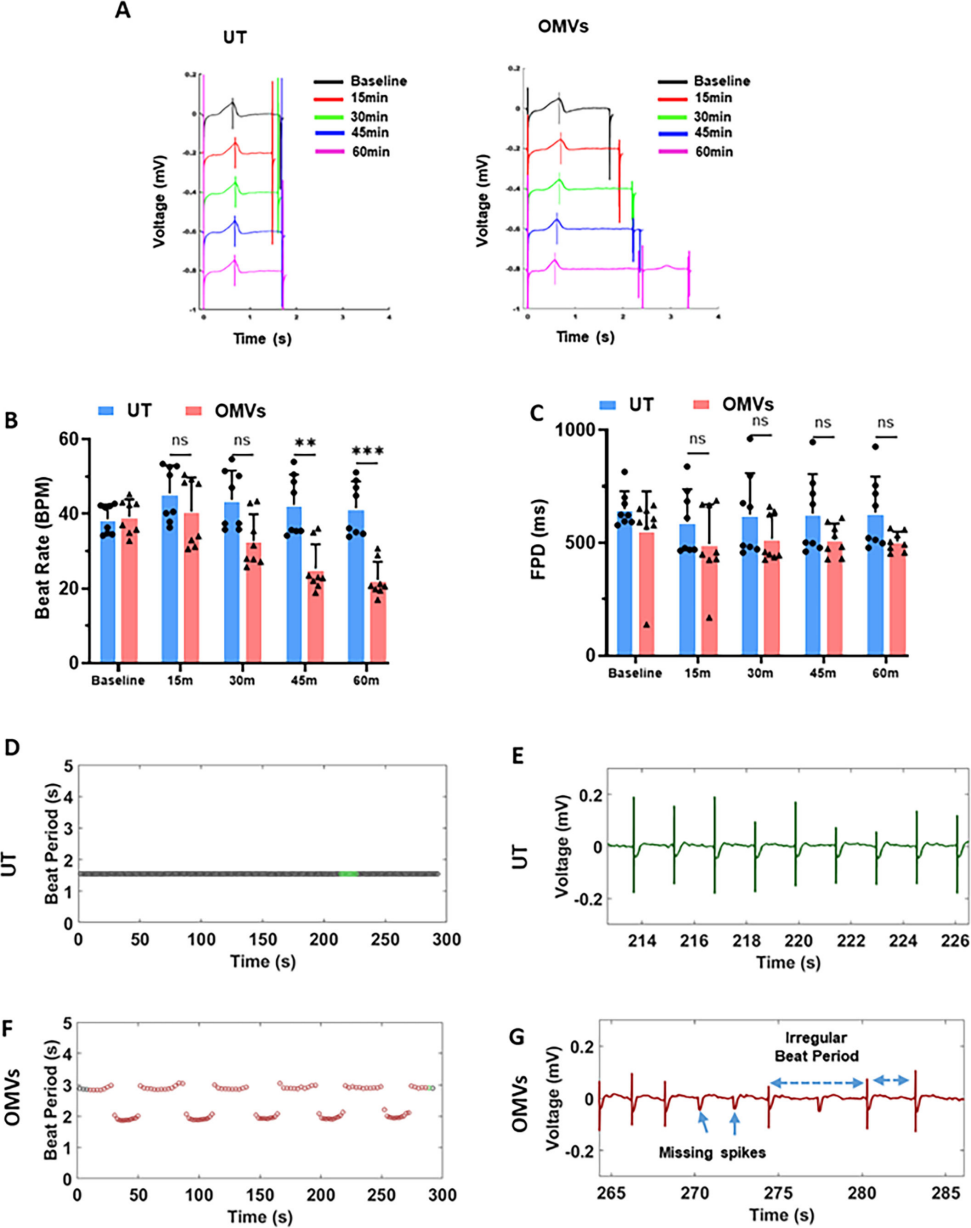
*P.a*. OMVs are potent inducers of cardiomyocyte contractile dysfunction. hiPSC-CMs were cultured on the MEA plate and exposed to OMVs or left untreated (UT), and the cardiomyocyte contractility and electrical activity were recorded using AxIS Navigator on the MEA system at 5% CO2 and at 37°C for indicated time points. (**A**) Representative traces recorded with the MEA system showing the beat period, T-wave, and FPD in UT and OMV-treated hiPSC-CMs. (**B**) Beats per minute and (**C**) FPD at baseline, 15, 30, 45, and 60 min post-treatment of hiPSC-CMs with OMVs. (**D–E**) Representative beat period of untreated (**F–G**) OMV-treated hiPSC-CMs over time. Data shown in panels B and C are representative of four independent experiments, mean ± SD: *P < 0.05, **P < 0.01, ***P < 0.001, ****P < 0.0001.

*P.a.* secretes bacterial products, including toxins and OMVs (4, 38). These OMVs are packed with bacterial toxins and antigens (38). To determine whether OMVs alone can induce cardiomyocyte contractile dysfunction, we isolated OMVs from *P.a.* culture media using published methods (38). We exposed hiPSC-CMs to OMVs (1:3.8 × 10^6^ ratio, hiPSC-CMs: OMVs) or left them untreated, and cardiomyocyte contractile function was monitored using MEA. Exposure of OMVs significantly increased beat period (Fig. 4A) and decreased beat rate (Fig. 4B) of hiPSC-CMs.

We next measured the FPD, representing the interval between depolarization and repolarization. OMV-treated cardiomyocytes exhibited a significant decrease in FPD compared to untreated cells (Fig. 4C). Additionally, OMV treatment caused irregular beat periods over a 300-second recording interval (Fig. 4D vs. F). Analysis of individual beats revealed that, unlike the regular intervals observed in untreated cells (Fig. 4E), OMV-treated cardiomyocytes displayed irregular beat intervals with missing depolarization spikes (Fig. 4G), demonstrating an arrhythmic phenotype similar to that induced by *P.a.* C-media treatment (Fig. 1F through I).

Because OMVs are enriched with proteinaceous virulence factors, such as β-lactamase, exotoxin A, Flagellin C, alkaline phosphatase, hemolytic phospholipase C, and polysaccharides including LPS and EPS (12, 39), we tested whether digesting these proteins with proteinase K could reverse the contractile dysfunction. OMVs were treated with proteinase K and applied to hiPSC-CMs (Fig. S8A). MEA recordings showed that untreated OMVs significantly reduced cardiomyocyte electrical activity, as indicated by lower beat rates at 1 hcompared to untreated controls (Fig. S8B and C). In contrast, cardiomyocytes exposed to proteinase K–treated OMVs exhibited improved electrical activity and significantly higher beat rates at 1 h. At later time points, cardiomyocytes treated with intact OMVs ceased contracting, whereas those exposed to proteinase K–digested OMVs maintained improved electrical function and higher beat rates (Fig. S8B and C). Together, these results indicate that OMV-induced cardiomyocyte contractile dysfunction is mediated by both protein-based and non-protein-based bacterial components.

### Conditioned medium from *P.a.*-infected hMDMs inhibits calcium transient cardiomyocytes and causes arrhythmia

**Fig 5:**
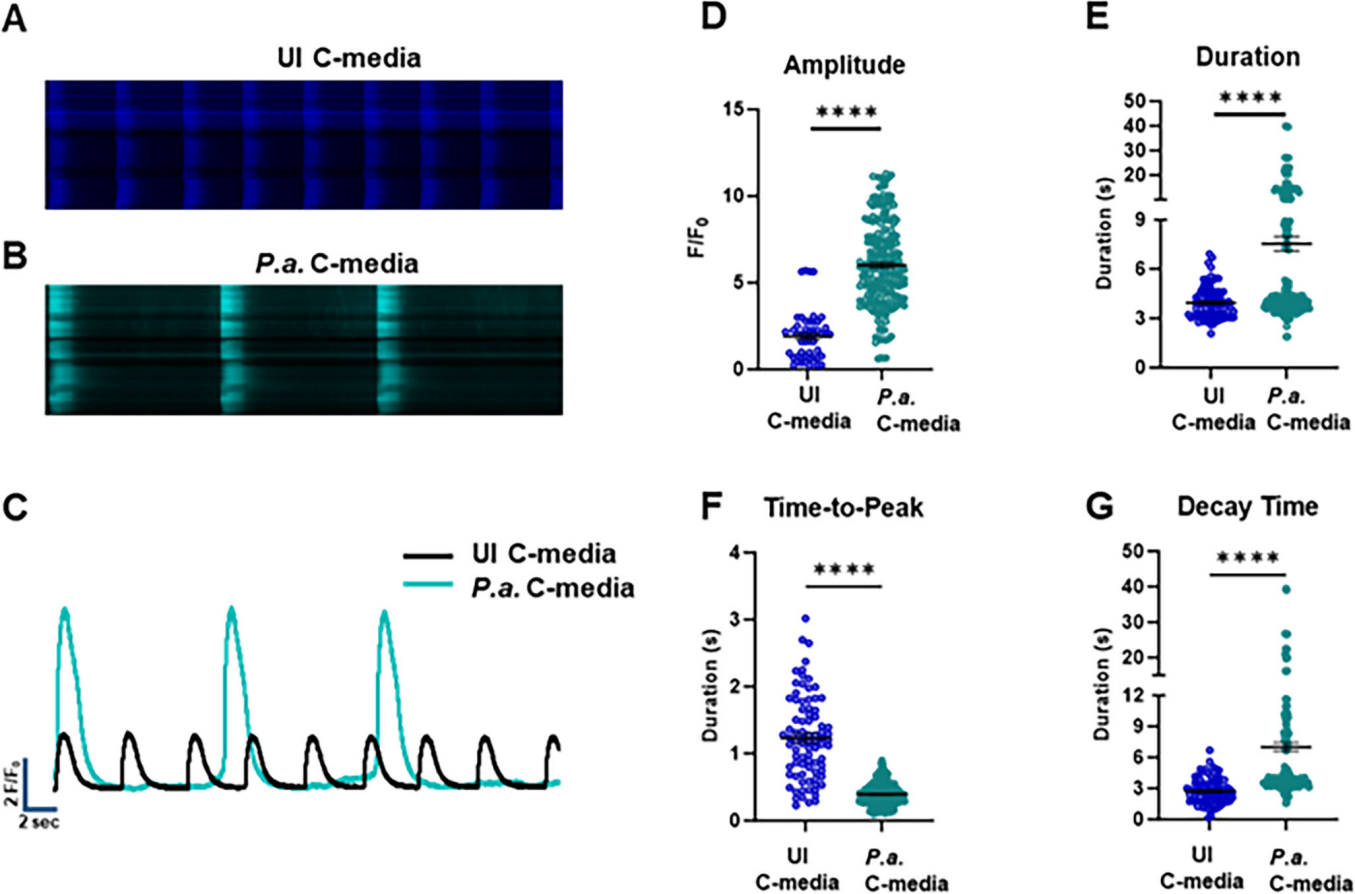
C-media from *P.a*.-infected hMDMs dysregulates calcium channel function in cardiomyocytes. Synchronized hiPSC-CMs were treated with UI-C-media or *P.a*. C-media, and the Ca²^+^ transient was visualized using Fluo-3AM. Representative images of Ca^2+^ transient wave of cardiomyocytes exposed to UI-C-media (**A**) and *P.a*. C-media (**B**). (**C**) Representative overlay of Ca^2+^ traces collected from cardiomyocytes incubated with UI-C-media and *P.a*. C-media. Ca^2+^ transient parameters, such as (**D**) peak amplitude, (**E**) peak duration, (**F**) time-to-peak duration, and (**G**) decay time, were measured in UI C-media and *P.a*. C-media-treated cardiomyocytes. Graphs shown are accumulative data acquired from 10 to 15 cells of three independent experiments; a two-tailed Student’s *t*-test was performed for comparison with control, **** *P* ˂ 0.0001.

Intracellular Ca^2+^ cycling is essential for maintaining cardiac rhythms, and disruptions in Ca^2+^ handling can lead to arrhythmias (40, 41). Since we observed that the C-media induces EAD and severe arrhythmias (Fig. 1; Fig. S4), we next examined its effects on intracellular Ca dynamics. hiPSC-CMs were stained with Fluo-3 AM to evaluate the intracellular Ca^2+^ transients in the presence of C-media harvested from *P.a.*-infected hMDMs, compared with the cardiomyocyte culture medium (Fig. 5A through C). Spontaneously beating cardiomyocytes exhibited a significant increase in peak Ca^2+^ amplitude within 30 min of C-media exposure compared with untreated controls (Fig. 5D). Similarly, C-media-treated hiPSC-CMs displayed irregular Ca^2+^ transient intervals, indicating disrupted synchronous beating (Fig. 5E). Moreover, C-media exposure reduced time-to-peak and markedly prolonged Ca^2+^ decay time (Fig. 5F and G). Collectively, these results suggest that components in C-media, potentially including EVs, impair Ca^2+^ cycling in hiPSC-CMs, thereby contributing to contractile dysfunction and arrhythmogenesis.

### EVs released from *P.a.*-infected hMDMs are packed with bacterial OMVs

**Fig 6:**
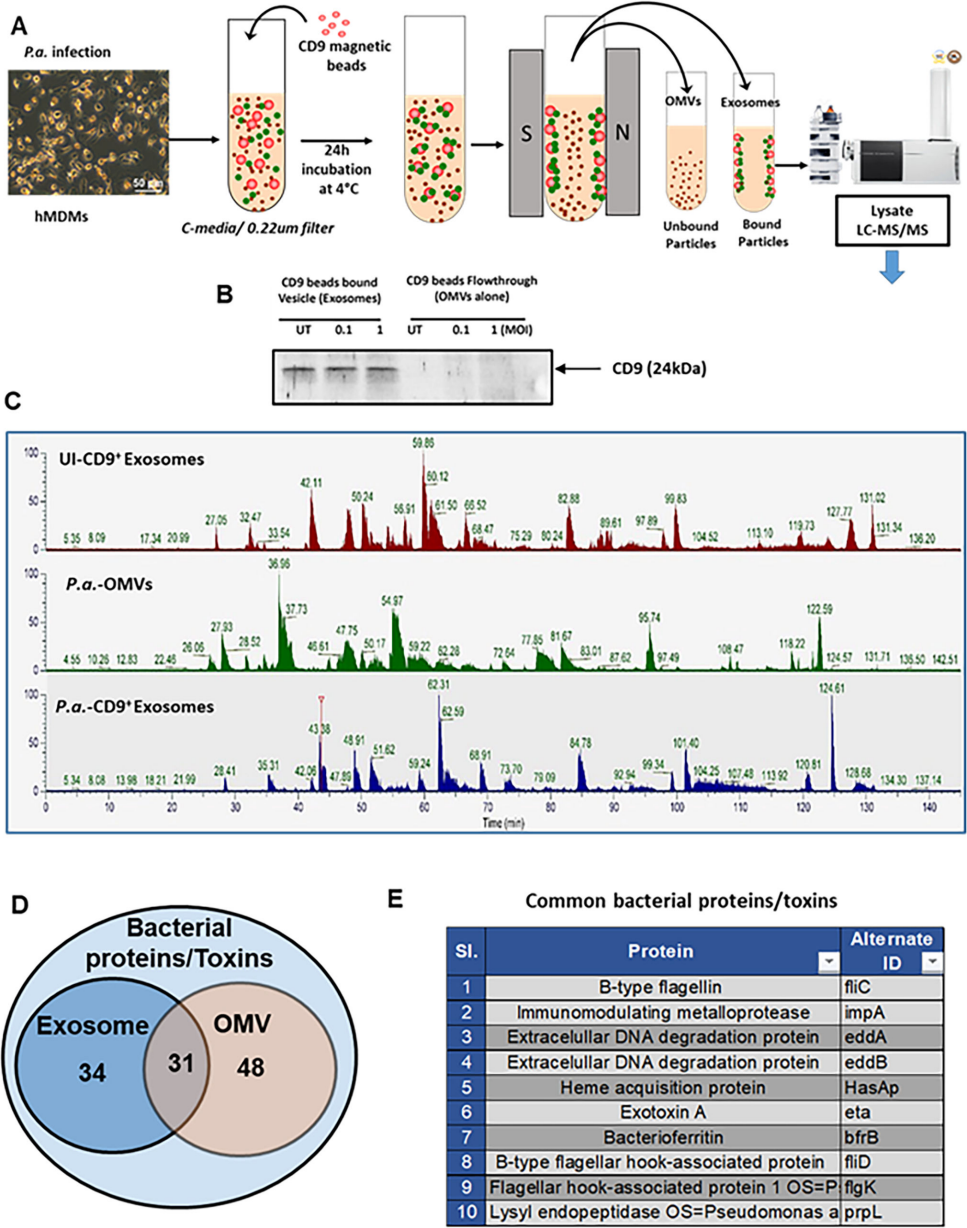
Exosomes from C-media contain bacterial antigens and products. Exosomes were harvested from uninfected and *P.a.*-infected hMDMs and subject to LC-MS/MS analysis. (**A**) Schematic explains the procedure for separating exosomes released from macrophages and *P.a.*-derived OMVs. (**B**) Western blot analysis of lysates from exosomes (CD9-bound) and OMV (flow-through) preparation; the membrane is probed with anti-CD9 antibody. (**C**) Spectral views of CD9^+^ exosomes from uninfected macrophages (top panel), purified OMVs (middle panel), and CD9^+^ exosomes from *P.a.*-infected macrophages (bottom panel). (**D**) The Venn diagram shows the bacterial proteins present in CD9^+^ exosomes alone, OMVs alone, or both. (**E**) The table lists the common bacterial antigens and toxins present in either CD9^+^ exosomes or OMVs.

Incubation of C-media harvested from *P.a.*-infected hMDMs caused cardiomyocyte contractile dysfunction (Fig. 1), and this C-media contained large numbers of EVs (Fig. 2E though G). Therefore, we next sought to characterize the contents of these EVs. EVs and OMVs were purified according to published methods (15, 38, 42), lysed with TN-1 lysis buffer, digested with trypsin, and analyzed by LC-MS/MS Orbitrap mass spectrometry. Peptide sequences were identified using the MASCOT database, and the resulting proteins are listed in Table 1. This analysis revealed several bacterial proteins within EVs isolated from *P.a.*-infected C-media, suggesting that EVs carry bacterial components, including immunogenic proteins, toxins, and cell wall elements that may contribute to cardiomyocyte contractile dysfunction. Since *P.a.* C-media contains both host-derived exosomes and bacterial OMVs, and our LC-MS/MS data indicated that bacterial proteins are present in these EVs, we next aimed to distinguish between these two populations. To achieve this, purified EVs were incubated with CD9 antibody-coated magnetic beads, which specifically bind to host-derived exosomes, allowing separation of exosomes and bacterial OMVs (schematic shown in Fig. 6A). The purity of the fractions was confirmed by western blot using CD9 antibody (Fig. 6B). To further identify their protein contents, CD9^+^ exosomes (from uninfected and infected hMDMs) and OMVs (from C-media of *P.a.*-infected hMDMs) were subjected to LC-MS/MS analysis. Comparative spectral profiling revealed several significant differences among the samples (Fig. 6C). Peptide sequences were analyzed against human and *P.a.* proteomic databases using MASCOT. We identified 65 unique bacterial proteins in the exosomes and 79 proteins in OMVs, with 31 bacterial proteins common to both vesicle types (Fig. 6D; Table 2). The top 10 common proteins (Fig. 6E) included bacterial proteases, toxins, and antigenic proteins known to modulate host cell signaling, suppress immune responses, and promote tissue damage (43–45). These include bacterial products, such as B-type flagellin, immunomodulating metalloproteases, endopeptidase, and toxins like exotoxin A. Collectively, these results indicate that *P. aeruginosa* infection triggers host cells to release both exosomes and OMVs that are enriched with bacterial proteins, which may underlie the observed cardiomyocyte contractile dysfunction.

**TABLE 1:** Proteins identified from exosomes harvested from uninfected (UI) and *P.a.*-infected (PAO1) hMDMs by LC-MS/MS^*a*^

SL	Name of bacterial protein	Accession number	Alternate ID	Mol. wt.	UI (unique peptide count)	*P.a.* (unique peptide count)
1	B-type flagellin	P72151	fliC	49 kDa	0	25
2	Type IV major pilin protein	P04739	pilA	16 kDa	0	9
3	Haemagg_act domain-containing protein	Q9I120	PA2462	573 kDa	0	86
4	Outer membrane porin F	P13794	oprF	38 kDa	0	15
5	Ferripyoverdine receptor	P48632	fpvA	91 kDa	0	28
6	Heme/hemoglobin uptake outer membrane receptor	Q9HV88	phuR	85 kDa	0	23
7	Probable hemagglutinin	Q9I791	PA0041	362 kDa	0	4
8	Exotoxin A	P11439	eta	69 kDa	0	23
9	Probable bacteriophage protein	G3XD39	PA0622	41 kDa	0	12
10	Secreted exoenzyme S	G3XDA1	exoS	48 kDa	0	20
11	Fe(3+)-pyochelin receptor	P42512	fptA	80 kDa	0	21
12	Type IV pilus biogenesis factor	Q9HVM8	pilY1	127 kDa	0	24
13	Elongation factor G1	Q9HWD2	fusA	78 kDa	0	18
14	Succinyltransferase 2-oxoglutarate dehydrogenase	Q9I3D2	sucB	43 kDa	0	12
15	Peptidoglycan-associated lipoprotein	Q9I4Z4	pal	18 kDa	0	6
16	Insulin-cleaving metalloproteinase outer membrane protein	Q9HW32	icmP	47 kDa	0	17
17	PvdL	Q9I157	pvdL	480 kDa	0	22
18	Major outer membrane lipoprotein	P11221	oprI	9 kDa	0	4
19	Chaperonin	P30718	groEL	57 kDa	0	13
20	ATP synthase subunit alpha	Q9HT18	atpA	55 kDa	0	13
21	Putative copper transport outer membrane porin	G3XD89	oprC	79 kDa	0	15
22	DNA-directed RNA polymerase subunit beta	Q51561	rpoB	151 kDa	0	19
23	Multidrug efflux outer membrane protein	Q9I0Y7	oprN	51 kDa	0	12
24	DUF3327 domain-containing protein	Q9I130	PA2452	34 kDa	0	8
25	LPS-assembly protein	Q9I5U2	lptD	104 kDa	0	16
26	Probable bacteriophage protein	G3XD71	PA0620	72 kDa	0	12
27	Probable bacteriophage protein	Q9I5S9	PA0623	18 kDa	0	4
28	B-type flagellar hook-associated protein 2	Q9K3C5	fliD	49 kDa	0	13
29	DNA-directed RNA polymerase subunit beta′	Q9HWC9	rpoC	154 kDa	0	16
30	Anaerobically induced outer membrane porin OprE	G3XDA5	oprE	50 kDa	0	9
31	Elongation factor Tu	P09591	tufA	43 kDa	0	10
32	ATP synthase subunit beta	Q9HT20	atpD	50 kDa	0	11
33	Fe (III) dicitrate transport protein	Q9HXB2	fecA	85 kDa	0	9
34	Efflux pump membrane transporter	Q9I0Y8	mexF	116 kDa	0	13
35	Multidrug efflux membrane fusion protein	Q9I0Y9	mexE	45 kDa	0	9
36	Exoenzyme T	Q9I788	exoT	49 kDa	0	12
37	Soluble pyridine nucleotide transhydrogenase	P57112	sthA	51 kDa	0	11
38	Probable TonB-dependent receptor	Q9HVC0	PA4675	81 kDa	0	11
39	NAD-specific glutamate dehydrogenase	Q9HZE0	gdhB	183 kDa	0	12
40	UPF0312 protein	Q9I690	PA0423	21 kDa	0	7
41	Flagellar hook protein	Q9I4P9	flgE	48 kDa	0	7
42	Arginine deiminase	P13981	arcA	46 kDa	0	9
43	L-ornithine N(5)-monooxygenase	Q51548	pvdA	49 kDa	0	9
44	Probable outer membrane protein	Q9HUJ1	PA4974	53 kDa	0	8
45	Polyribonucleotide nucleotidyltransferase	Q9HV59	pnp	75 kDa	0	10
46	Lipid A deacylase	Q9HVD1	pagL	18 kDa	0	6
47	Probable non-ribosomal peptide synthetase	Q9I179	PA2402	569 kDa	0	11
48	Ferric enterobactin receptor	Q9I527	pirA	81 kDa	0	9
49	Immunomodulating metalloprotease	Q9I5W4	impA	100 kDa	0	9
50	Ornithine carbamoyltransferase, catabolic	P08308	arcB	38 kDa	0	8
51	50S ribosomal protein L21	Q9HVL6	rplU	12 kDa	0	5
52	Second ferric pyoverdine receptor	Q9HWL3	fpvB	87 kDa	0	9
53	Rick 17kDa Anti domain-containing protein	Q9I4S1	PA1053	16 kDa	1	4
54	Serralysin	Q03023	aprA	50 kDa	0	7
55	Acetyltransferase component of pyruvate dehydrogenase complex	Q59638	aceF	57 kDa	0	9
56	Outer membrane protein assembly factor BamB	Q9HXJ7	bamB	40 kDa	0	8
57	Pyoverdine synthetase D	Q9I182	pvdD	274 kDa	0	8
58	Flagellar hook-associated protein 1	Q9I4P3	flgK	72 kDa	0	9
59	AmpDh3	Q9I5D1	ampDh3	29 kDa	0	5
60	Tol-Pal system protein	P50601	tolB	48 kDa	0	8
61	Chaperone protein	Q9HV43	dnaK	68 kDa	0	8
62	Type 4 fimbrial biogenesis protein	Q9HVM9	pilX	21 kDa	0	6
63	Uncharacterized protein	Q9HVZ2	PA4423	66 kDa	0	7
64	Peptidase M75 domain-containing protein	Q9HW30	PA4372	38 kDa	0	5
65	Outer membrane protein	Q9HWW1	oprG	25 kDa	0	5
66	Oxoglutarate dehydrogenase (succinyl-transferring)	Q9I3D3	sucA	106 kDa	0	8
67	Alkyl hydroperoxide reductase C	Q9I6Z3	ahpC	21 kDa	0	8
68	Uncharacterized protein	G3XD83	PA0625	78 kDa	0	6
69	30S ribosomal protein S4	O52759	rpsD	23 kDa	0	6
70	Aspartate—tRNA (Asp/Asn) ligase	Q51422	aspS	66 kDa	0	6
71	Translation initiation factor IF-2	Q9HV55	infB	91 kDa	0	6
72	50S ribosomal protein L25	Q9HVC4	rplY	22 kDa	0	4
73	Glutamate dehydrogenase	Q9HVJ7	gdhA	49 kDa	0	6
74	50S ribosomal protein L2	Q9HWD8	rplB	30 kDa	0	6
75	30S ribosomal protein S3	Q9HWE1	rpsC	26 kDa	0	4
76	6,7-dimethyl-8-ribityllumazine synthase	Q9HWX5	ribH	16 kDa	0	4
77	30S ribosomal protein S1	Q9HZ71	rpsA	62 kDa	0	6
78	Fatty acid oxidation complex subunit alpha	Q9HZJ2	fadB	77 kDa	0	7
79	Probable outer membrane protein	Q9I083	PA2760	47 kDa	0	6
80	PvdJ	Q9I181	pvdJ	240 kDa	0	4
81	PhoP/Q and low Mg2+ inducible outer membrane protein H1	G3XD11	oprH	22 kDa	0	5
82	Serine-type D-Ala-D-Ala carboxypeptidase	G3XD74	dacC	42 kDa	0	4
83	Citrate synthase	P14165	gltA	48 kDa	0	5
84	Co-chaperonin	P30720	groES	10 kDa	0	4
85	Fimbrial assembly protein	P34750	pilQ	77 kDa	0	6
86	Fumarate hydratase class II	Q51404	fumC2	49 kDa	0	5
87	Outer membrane protein	Q51487	oprM	53 kDa	0	6
88	ATP synthase gamma chain	Q9HT19	atpG	32 kDa	0	5
89	Uncharacterized protein	Q9HVY9	PA4426	21 kDa	0	6
90	30S ribosomal protein S7	Q9HWD1	rpsG	18 kDa	0	4
91	Heme uptake outer membrane receptor	Q9HYJ7	hasR	98 kDa	0	5
92	Probable TonB-dependent receptor	Q9HYX3	PA3268	79 kDa	0	6
93	Isocitrate lyase	Q9I0K4	PA2634	59 kDa	0	6
94	Isocitrate dehydrogenase (NADP)	Q9I0L5	icd	46 kDa	0	6
95	Pyoverdine biosynthesis protein	Q9I183	pvdE	61 kDa	0	6
96	Dihydrolipoyl dehydrogenase	Q9I3D1	lpdG	50 kDa	0	6
97	Uncharacterized protein	G3XCU8	PA0628	36 kDa	0	4
98	Probable bacteriophage protein	G3XCX5	PA0618	32 kDa	0	5
99	DNA-directed RNA polymerase subunit alpha	O52760	rpoA	37 kDa	0	4
100	30S ribosomal protein S2	O82850	rpsB	27 kDa	0	4
101	Porin D	P32722	oprD	48 kDa	0	4
102	Outer membrane protein assembly factor BamD	P33641	bamD	39 kDa	0	5
103	Succinate—CoA ligase subunit beta	P53593	sucC	42 kDa	0	5
104	AsmA domain-containing protein	Q9HU38	PA5146	81 kDa	0	5
105	Glutamine synthetase	Q9HU65	glnA	52 kDa	0	5
106	Malic enzyme	Q9HUD3	PA5046	45 kDa	0	4
107	Protein HflC	Q9HUM3	hflC	33 kDa	0	5
108	50S ribosomal protein L1	Q9HWC6	rplA	24 kDa	0	4
109	Uncharacterized protein	Q9HXR3	PA3729	76 kDa	0	5
110	Outer membrane protein assembly factor	Q9HXY4	opr86	88 kDa	0	5
111	Probable dipeptidase	Q9I187	PA2393	49 kDa	0	5
112	Probable outer membrane protein	Q9I189	opmQ	51 kDa	0	4
113	Phosphoenolpyruvate synthase	Q9I2W9	ppsA	86 kDa	0	5
114	Succinate dehydrogenase (B subunit)	Q9I3D4	sdhB	26 kDa	0	5
115	Probable binding protein component of ABC transporter	Q9I402	PA1342	33 kDa	0	4
116	Flagellar basal-body rod protein	Q9I4P7	flgG	28 kDa	0	4
117	S-adenosylmethionine synthase	Q9I5Z0	metK	43 kDa	0	4
118	Gly-zipper_Omp domain-containing protein	Q9I762	PA0070	32 kDa	0	5
119	Type 4 fimbrial biogenesis protein	G3XCZ0	fimU	18 kDa	0	3
120	Uncharacterized protein	Q9HVF2	PA4639	21 kDa	0	3
121	Lysosomal acid phosphatase	P11117	ACP2	48 kDa	0	3
122	Chaperone SurA	Q9I5U3	surA	47 kDa	0	3
123	Probable outer membrane receptor for iron transport	G3XCY8	PA4514	82 kDa	0	4
124	Type III secretion protein	G3XD49	pcrV	32 kDa	0	4
125	Multidrug resistance protein	P52002	mexB	113 kDa	0	4
126	ATP synthase subunit b	Q9HT16	atpF	17 kDa	0	4
127	50S ribosomal subunit assembly factor	Q9HU67	typA	67 kDa	0	4
128	Protein HflK	Q9HUM2	hflK	44 kDa	0	4
129	Uncharacterized protein	Q9HUW8	PA4842	40 kDa	0	4
130	DUF4136 domain-containing protein	Q9HV15	PA4793	21 kDa	0	4
131	Ketol-acid reductoisomerase (NADP(+))	Q9HVA2	ilvC	36 kDa	0	4
132	Serine hydroxymethyltransferase 3	Q9HVI7	glyA2	45 kDa	0	4
133	Energy-dependent translational throttle protein	Q9HVJ1	ettA	61 kDa	0	4
134	Lipopolysaccharide export system protein	Q9HVV7	lptA	19 kDa	0	4
135	Methyl-accepting chemotaxis protein	Q9HW91	pctB	68 kDa	0	4
136	50S ribosomal protein L10	Q9HWC7	rplJ	18 kDa	0	3
137	50S ribosomal protein L3	Q9HWD5	rplC	23 kDa	0	4
138	50S ribosomal protein L18	Q9HWF1	rplR	13 kDa	0	4
139	LPS-assembly lipoprotein	Q9H X 32	lptE	23 kDa	0	4
140	Inosine-5′-monophosphate dehydrogenase	Q9HXM5	guaB	52 kDa	0	4
141	Probable malate:quinone oxidoreductase 1	Q9HYF4	mqo1	57 kDa	0	4
142	Elongation factor P	Q9HZZ2	efp	21 kDa	0	4
143	ATP-dependent RNA helicase	Q9I003	deaD	62 kDa	0	4
144	Uncharacterized protein	Q9I131	PA2451	22 kDa	0	3
145	L-2,4-diaminobutyrate:2-ketoglutarate 4-aminotransferase	Q9I168	pvdH	50 kDa	0	4
146	PvdR	Q9I191	pvdR	42 kDa	0	4
147	Peptidyl-prolyl cis-trans isomerase D	Q9I2T8	ppiD	69 kDa	0	4
148	Trigger factor	Q9I2U2	tig	49 kDa	0	4
149	Flagellar hook-associated protein type 3	Q9I4P2	flgL	47 kDa	0	4
150	Uncharacterized protein	Q9I503	PA0955	36 kDa	0	3
151	Probable acyl-CoA dehydrogenase	Q9I612	PA0506	65 kDa	0	4
152	Type VI secretion system spike protein	Q9I737	vgrG1b	83 kDa	0	4
153	30S ribosomal protein S11	Q9HWF8	rpsK	14 kDa	0	3
154	Bacterioferritin	Q9HY79	bfrB	19 kDa	0	4
155	30S ribosomal protein S21	Q9I5V8	rpsU	8 kDa	0	4
156	Probable cold-shock protein	Q9I662	PA0456	8 kDa	0	3
157	Elongation factor Ts	O82851	tsf	31 kDa	0	3
158	Uncharacterized protein	Q9I129	PA2453	8 kDa	0	3
159	50S ribosomal protein L4	Q9HWD6	rplD	22 kDa	0	3
160	ATP synthase subunit delta	Q9HT17	atpH	19 kDa	0	3
161	50S ribosomal protein L6	Q9HWF0	rplF	19 kDa	0	3
162	ATP synthase subunit a	Q9HT14	atpB	32 kDa	0	2
163	50S ribosomal protein L22	Q9HWE0	rplV	12 kDa	0	2
164	Ribonuclease E	Q9HZM8	rne	117 kDa	0	2
165	Acyl-coenzyme A dehydrogenase	Q9I028	PA2815	89 kDa	1	2
166	Succinate—CoA ligase subunit alpha	Q51567	sucD	30 kDa	0	3
167	RNA-binding protein	Q9HUM0	hfq	9 kDa	0	3
168	Cell shape-determining protein	Q9HVU0	mreB	37 kDa	0	3
169	50S ribosomal protein L11	Q9HWC5	rplK	15 kDa	0	3
170	Protein translocase subunit	Q9HXI1	secD	68 kDa	0	3
171	Usp domain-containing protein	Q9HYT5	PA3309	16 kDa	0	3
172	Uncharacterized protein	Q9I1A2	PA2377	47 kDa	0	3
173	Probable siderophore receptor	Q9I3 X 9	PA1365	89 kDa	0	3
174	Uncharacterized protein	Q9I4W2	PA1011	43 kDa	0	3
175	Cytosine deaminase	Q9I680	codA	47 kDa	0	3
176	Lipoprotein NlpD/LppB homolog	P45682	PA3623	31 kDa	0	3
177	GP-PDE domain-containing protein	Q9HV16	PA4792	34 kDa	0	3
178	Alpha-2-macroglobulin homolog	Q9HVT2	PA4489	167 kDa	0	3
179	Probable transporter	Q9HWG8	PA4218	43 kDa	0	3
180	Peptidyl-prolyl cis-trans isomerase	Q9HYX8	PA3262	27 kDa	0	3
181	Electron transfer flavoprotein subunit alpha	Q9HZP7	etfA	31 kDa	0	3
182	Probable aminotransferase	Q9I0V2	PA2531	41 kDa	0	3
183	Membrane protein insertase	Q9HT06	yidC	64 kDa	0	3
184	LysM domain-containing protein	Q9HUP0	PA4924	25 kDa	0	3
185	Uncharacterized protein	Q9I043	PA2800	26 kDa	0	3
186	Ferrioxamine receptor	Q9I116	foxA	90 kDa	0	3
187	Adenosylhomocysteinase	Q9I685	ahcY	51 kDa	0	3
188	Uncharacterized protein	Q9HW14	PA4390	38 kDa	0	3
189	Uncharacterized protein	G3XCU0	PA1245	41 kDa	0	3
190	DNA-binding protein HU-beta	P05384	hupB	9 kDa	0	3
191	Threonine—tRNA ligase	Q9I099	thrS	73 kDa	0	3
192	Uncharacterized protein	Q9I683	PA0434	81 kDa	0	3
193	Alkyl hydroperoxide reductase C	Q9HY81	PA3529	22 kDa	0	3
194	Azurin	P00282	azu	16 kDa	0	3
195	Beta sliding clamp	Q9I7C4	dnaN	41 kDa	0	3
196	Uncharacterized protein	Q9HTT7	PA5258	41 kDa	0	3
197	Component of ABC iron transporter	Q9HTX3	PA5217	36 kDa	0	3
198	LysM domain-containing protein	Q9I7A7	PA0020	38 kDa	0	3
199	Uncharacterized protein	Q9HV97	PA4699	26 kDa	0	2
200	30S ribosomal protein S9	Q9HVY3	rpsI	15 kDa	0	2
201	30S ribosomal protein S12	Q9HWD0	rpsL	14 kDa	0	2
202	30S ribosomal protein S13	Q9HWF7	rpsM	13 kDa	0	2
203	Glucose dehydrogenase	Q9I1I5	gcd	86 kDa	0	2
204	Type IV pilus non-core minor pilin	G3XD43	pilE	15 kDa	0	2
205	PvdP	Q9I188	pvdP	62 kDa	0	2
206	AmpDh2	Q9HT86	ampDh2	29 kDa	0	2
207	Heme acquisition protein	G3XD33	hasAp	21 kDa	0	2
208	50S ribosomal protein	Q9HWE7	rplE	20 kDa	0	2
209	50S ribosomal protein L20	Q9I0A2	rplT	13 kDa	0	2
210	Peptidyl-prolyl cis-trans isomerase	Q9I2U9	ppiB	18 kDa	0	2
211	Chaperone protein	Q9I3C5	htpG	72 kDa	0	2
212	Major cold shock protein	P95459	cspA	8 kDa	0	2
213	Uncharacterized protein	G3XD65	PA0626	31 kDa	0	2
214	Probable short-chain dehydrogenase	Q9HW15	speA	27 kDa	0	2
215	Protein	P42257	pilJ	73 kDa	0	2
216	UDP-3-O-acyl-N-acetylglucosamine deacetylase	P47205	lpxC	33 kDa	0	2
217	D-amino acid dehydrogenase 1	Q9HTQ0	dadA1	47 kDa	0	2
218	Probable carboxyl-terminal protease	Q9HU50	PA5134	46 kDa	0	2
219	Osmotically inducible lipoprotein	Q9HUT7	osmE	13 kDa	0	2
220	DUF4398 domain-containing protein	Q9HZU6	PA2901	13 kDa	0	2
221	ATP-dependent Clp protease proteolytic subunit 1	Q9I2U1	clpP1	24 kDa	0	2
222	Chitin-binding protein	Q9I589	cbpD	42 kDa	0	2
223	Bifunctional protein	Q9I5F6	putA	116 kDa	0	2
224	Transketolase	Q9I5Y8	tktA	72 kDa	0	2
225	Autotransporter domain-containing protein	Q9I6G3	PA0328	70 kDa	0	2
226	Protein translocase subunit	Q9LCT3	secA	104 kDa	0	2
227	Uncharacterized protein	Q9I752	PA0080	17 kDa	0	2
228	Transcription elongation factor	Q9HV46	greA	17 kDa	0	2
229	Ribose-phosphate pyrophosphokinase	Q9HVC5	prs	34 kDa	0	2
230	Peptidase_M48 domain-containing protein	Q9HVF9	PA4632	29 kDa	0	2
231	Uncharacterized protein	Q9HXG8	PA3836	34 kDa	0	2
232	Uncharacterized protein	Q9I2D5	PA1969	13 kDa	0	2
233	Pyochelin synthetase	Q9HWG4	pchF	197 kDa	0	2
234	Nucleoside diphosphate kinase	Q59636	ndk	16 kDa	0	2
235	Probable carbamoyl transferase	Q9HUG0	PA5005	66 kDa	0	2
236	50S ribosomal protein L28	Q9HTN8	rpmB	nine kDa	0	2
237	Penicillin-binding protein 1A	Q07806	mrcA	91 kDa	0	2
238	Ferredoxin—NADP reductase	Q9HYK7	fpr	30 kDa	0	2
239	GMP synthase	Q9HXM6	guaA	58 kDa	0	2
240	Haemagg_act domain-containing protein	Q9HVG6	PA4625	220 kDa	0	2
241	Type 4 fimbrial biogenesis protein	G3XD84	pilV	20 kDa	0	2
242	Ubiquinol-cytochrome c reductase	Q9HVY4	PA4431	21 kDa	0	2
243	Uncharacterized protein	Q9I2 X 2	PA1767	57 kDa	0	2
244	Leucine, isoleucine	P21175	braC	40 kDa	0	2
245	Carbamoyl-phosphate synthase large chain	P38100	carB	117 kDa	0	2
246	Phosphoenolpyruvate carboxykinase (ATP)	Q9HTZ7	pckA	56 kDa	0	2
247	Fructose-bisphosphate aldolase	Q9I5Y1	fba	39 kDa	0	2
248	Probable cytochrome c1	Q9HVY6	PA4429	29 kDa	0	2
249	Probable enoyl-CoA hydratase/isomerase	Q9I5I4	PA0745	30 kDa	0	2
250	50S ribosomal protein L7/L12	Q9HWC8	rplL	12 kDa	0	2
251	Probable acyl-CoA thiolase	Q9I0T1	PA2553	41 kDa	0	2
252	Multidrug resistance protein	P52477	mexA	41 kDa	0	2
253	Glucose-6-phosphate isomerase	Q9HV67	pgi	62 kDa	0	2
254	NMO domain-containing protein	Q9I5R1	PA0660	35 kDa	0	2
255	Glycine betaine transmethylase	Q9HZC6	gbt	71 kDa	0	2
256	Motility hub protein	Q9HZA6	fimV	97 kDa	0	2
257	Arginine—tRNA ligase	Q9HUC8	argS	65 kDa	0	2
258	Probable bacteriophage protein	Q9I5S5	PA0641	131 kDa	0	2
259	Fructose-1,6-bisphosphatase class 1	Q9HU73	fbp	37 kDa	0	2
260	N-acetylmuramoyl-L-alanine amidase	Q9HUL7	amiB	51 kDa	0	2
261	Tol-Pal system protein	P50598	tolQ	25 kDa	0	2
262	Malate synthase G	Q9I636	glcB	79 kDa	0	2
263	Secretin	P35818	xcpQ	70 kDa	0	2
264	Uncharacterized protein	Q9HXR1	PA3731	25 kDa	0	2
265	Lysyl endopeptidase	Q9HWK6	prpL	48 kDa	0	2
266	Esterase	O33407	estA	70 kDa	0	2
267	Xenobiotic reductase	Q9HW45	xenB	38 kDa	0	2
268	Isocitrate dehydrogenase	Q9I0L4	idh	82 kDa	0	2
269	Uncharacterized protein	Q9I082	PA2761	16 kDa	0	2
270	Probable oxidoreductase	Q9I0Z1	PA2491	37 kDa	0	2
271	TPR repeat-containing protein	P42810	PA4667	66 kDa	0	2
272	30S ribosomal protein S19	Q9HWD9	rpsS	10 kDa	0	2
273	Glyceraldehyde-3-phosphate dehydrogenase	Q9HZK4	PA3001	50 kDa	0	2
274	Protein translocase subunit	Q9HWF5	secY	48 kDa	0	2
275	RNA polymerase sigma factor	P26480	rpoD	70 kDa	0	2
276	OmpA-like domain-containing protein	Q9I5A7	PA0833	25 kDa	0	2
277	Probable malate:quinone oxidoreductase 2	Q9HVF1	mqo2	55 kDa	0	2
278	Lipopolysaccharide biosynthetic protein	Q9I522	lpxO2	36 kDa	0	2
279	Protein translocase subunit	Q9HXI2	secF	33 kDa	0	2
280	50S ribosomal protein L13	Q9HVY2	rplM	16 kDa	0	2
281	Rick_17kDa_Anti domain-containing protein	Q9HXI3	PA3819	19 kDa	0	2
282	ABC_trans_aux domain- protein	Q9HUK2	PA4963	26 kDa	0	2
283	Probable DNA-binding protein	Q9H X 76	PA3940	10 kDa	0	2
284	Thioredoxin peroxidase	Q9I4W5	bcp	17 kDa	0	2
285	Probable protein kinase	Q9HUB8	ubiB	62 kDa	0	2
286	Pyruvate dehydrogenase E1 component	Q59637	aceE	100 kDa	0	2
287	Alanine—tRNA ligase	Q9I553	alaS	95 kDa	0	2
288	Histidine kinase	Q9I696	chpA	269 kDa	0	2
289	GDP-polyphosphate phosphotransferase	Q9I6Z1	ppk2	41 kDa	0	2
290	Cyclohexadienyl dehydratase	Q01269	pheC	30 kDa	0	2
291	Proline—tRNA ligase	Q9I502	proS	63 kDa	0	2
292	50S ribosomal protein	Q9HWE5	rplN	13 kDa	0	2

^*a*^ Overnight cultured hMDMs were infected with 1 MOI of *P.a.* for 22 h, and the cell-free culture supernatants were used to harvest the EVs using the above-described protocol (Materials and Methods). An equal amount of EV proteins was subjected to LC-MS/MS, and data were analyzed using Scaffold version 5.2.0. The unique bacterial proteins found in the EVs were listed based on the presence of unique peptide score.

**TABLE 2:** Bacterial proteins that are common in both exosomes and OMVs^*a*^

SL#	Proteins	Accession number	Alternate ID	Normalized total spectra (exosomes)	Normalized total spectra (OMVs)
1	OX=208,964 GN=fliC PE=1 SV=2	P72151	fliC	237.06	41.889
2	OX=208,964 GN=phoA PE=1 SV=2	P35483	phoA	135.09	107.55
3	OX=208,964 GN=PA0623 PE=1 SV=1	Q9I5S9	PA0623	112.57	156.23
4	OX=208,964 GN=PA0622 PE=1 SV=1	G3XD39	PA0622	30.46	107.55
5	OX=208,964 GN=groEL PE=1 SV=3	P30718	groEL	22.514	49.814
6	OX=208,964 GN=fliD PE=1 SV=1	Q9K3C5	fliD	18.541	15.85
7	OX=208,964 GN=glpQ PE=4 SV=1	Q9I6E6	glpQ	18.541	7.9249
8	OX=208,964 GN=PA0423 PE=1 SV=1	Q9I690	PA0423	11.919	22.643
9	OX=208,964 GN=PA0626 PE=1 SV=1	G3XD65	PA0626	11.919	15.85
10	OX=208,964 GN=eddA PE=4 SV=1	Q9HXA3	eddA	11.919	11.321
11	OX=208,964 GN=PA0628 PE=1 SV=1	G3XCU8	PA0628	9.2706	14.718
12	OX=208,964 GN=flgK PE=3 SV=1	Q9I4P3	flgK	9.2706	9.0571
13	OX=208,964 GN=pepA PE=3 SV=1	O68822	pepA	9.2706	4.5285
14	OX=208,964 GN=sthA PE=1 SV=2	P57112	sthA	6.6218	19.246
15	OX=208,964 GN=impA PE=1 SV=1	Q9I5W4	impA	6.6218	24.907
16	OX=208,964 GN=aceF PE=2 SV=2	Q59638	aceF	5.2975	6.7928
17	OX=208,964 GN=prpL PE=1 SV=1	Q9HWK6	prpL	3.9731	15.85
18	OX=208,964 GN=sucC PE=1 SV=2	P53593	sucC	3.9731	5.6607
19	OX=208,964 GN=hasAp PE=1 SV=1	G3XD33	hasAp	3.9731	4.5285
20	OX=208,964 GN=PA2635 PE=4 SV=1	Q9I0K3	PA2635	3.9731	1.1321
21	OX=208,964 GN=lpdG PE=1 SV=1	Q9I3D1	lpdG	2.6487	7.9249
22	OX=208,964 GN=PA0641 PE=4 SV=1	Q9I5S5	PA0641	2.6487	1.1321
23	OX=208,964 GN=PA3529 PE=1 SV=1	Q9HY81	PA3529	2.6487	3.3964
24	OX=208,964 GN=rpoC PE=3 SV=1	Q9HWC9	rpoC	2.6487	1.1321
25	OX=208,964 GN=braC PE=1 SV=2	P21175	braC	1.3244	10.189
26	OX=208,964 GN=azu PE=1 SV=2	P00282	azu	1.3244	11.321
27	OX=208,964 GN=eddB PE=4 SV=1	Q9HXA4	eddB	1.3244	4.5285
28	OX=208,964 GN=arcA PE=1 SV=2	P13981	arcA	1.3244	3.3964
29	OX=208,964 GN=pnp PE=3 SV=1	Q9HV59	pnp	1.3244	3.3964
30	OX=208,964 GN=PA5505 PE=1 SV=1	Q9HT68	PA5505	1.3244	3.3964
31	OX=208,964 GN=guaB PE=1 SV=1	Q9HXM5	guaB	1.3244	2.2643

^*a*^ Overnight cultured hMDMs were infected with 1 MOI of *P.a.* for 22 h; the cell-free culture supernatants were used to harvest the EVs using the above-described protocol (Materials and Methods). To separate the bacterial OMVs and host-derived exosomes, we incubated the EVs with antiCD9 antibody-coated beads and separated the exosomes and OMVs using magnetic columns. The purified exosomes and OMVs were subjected to LC-MS/MS analysis. The unique bacterial proteins found in both OMVs and exosomes are listed in the table.

To further validate our LC-MS/MS findings, lysates from CD9^+^ exosomes and CD9^-^ OMVs separated from total EVs were analyzed by western blot using anti-flagellin-B antibody. Consistent with our proteomic data, flagellin-B was detected in both exosomes and OMVs, confirming that EV preparations contain both host-derived exosomes loaded with bacterial proteins and bacterial OMVs (Fig. S9A). Next, we isolated exosomes from bronchoalveolar lavage fluid (BALF) of control and *P.a.*-infected mice and examined them for the presence of flagellin-B. Exosomes from the BALF of *P.a*.-infected mice contained detectable flagellin-B (Fig. S9B). Since *P.a.* infection is common and associated with severe cardiovascular complications (46, 47) in patients admitted to intensive care units (ICUs), we further examined exosomes from the serum of healthy donors and *P. aeruginosa* culture-positive ICU patients. Notably, exosomes from ICU patients with confirmed *P.a.* infection contained flagellin-B at varying levels (Fig. S9C). Together, these findings strongly support that exosomes released during *P. aeruginosa* infection in both mice and humans carry bacterial antigens and toxins, which may contribute to infection-associated cardiac dysfunction.

### *P.a.* OMVs cause cardiac dysfunction and increase mortality in mice

**Fig 7:**
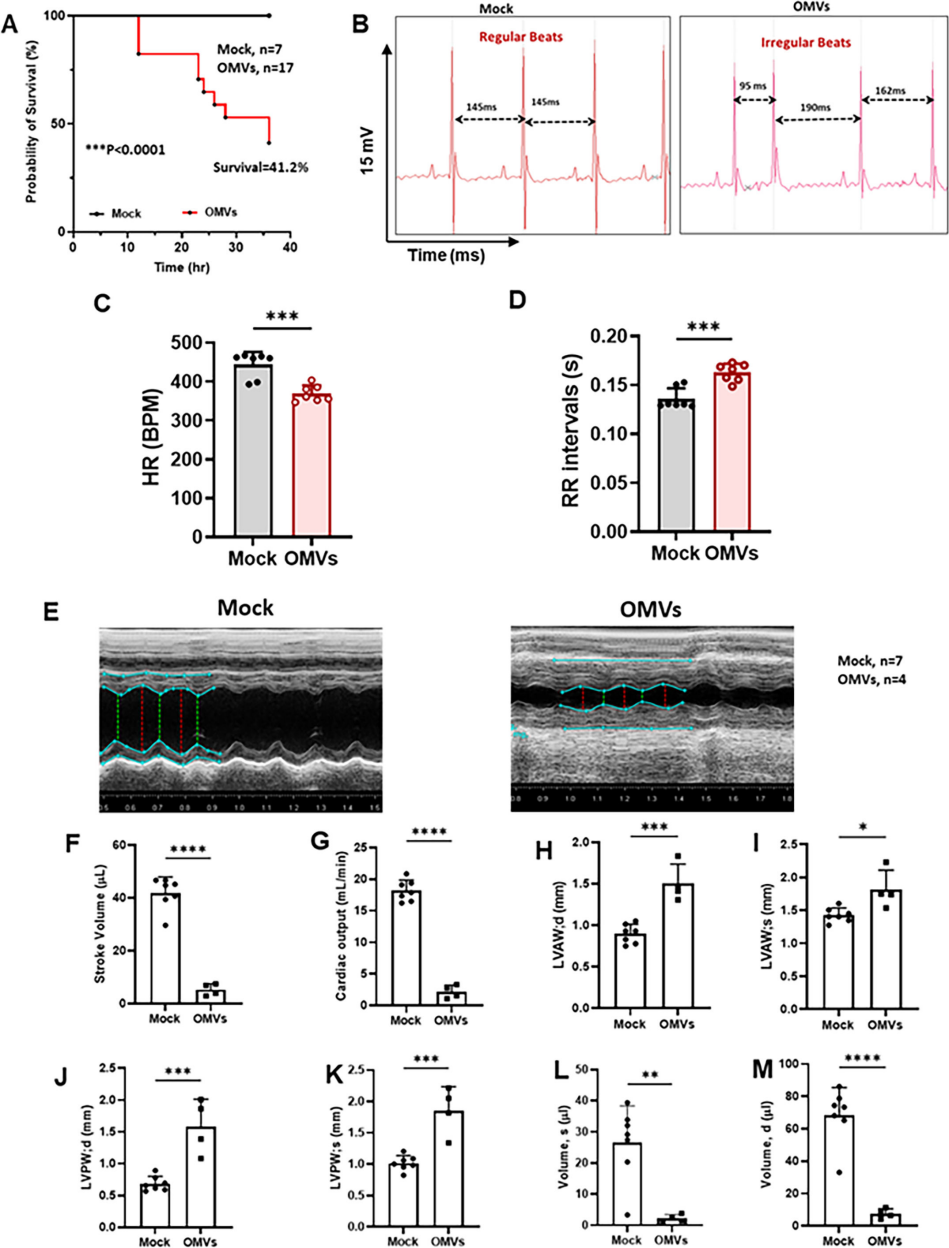
OMVs from *P.a.* cause cardiac electrical, LV dysfunction, and mortality in mice. C57BL/6 mice were injected intravenously with *P.a.* OMVs (10 mg/kg body weight) or saline, and survival of the mice was monitored for 36 h. (**A**) The graph shows the survival rate of mice infused with OMVs (*N* = 17), cumulative data from two experiments. (**B**) Representative ECG traces from mock and OMV-infused mice; double head arrows indicate RR intervals. ECG traces were analyzed using LabChart 8 Pro (ADInstruments). (**C**) Heart rate and (**D**) RR intervals. To assess heart function *in vivo*, 2D echocardiography (Vevo 2100, VisualSonics) was performed at 24 h post-OMV administration. (**E**) Representative images of cardiac patches from mock and OMV-administered mice. At least three M-mode echocardiogram measurements from each mouse were used to determine the (**F**) stroke volume, (**G**) cardiac output, (**H–I**) anterior, (**J–K**) posterior LV wall thickness, and (**L–M**) LV chamber volume during diastole and systole. The data shown are representative of two different experiments (Mock, *n* = 7; OMVs, *n* = 4), mean ± SD: **P < 0.01; **P < 0.001; ***P < 0.0001.

We previously demonstrated that *P.a.* infection induces cardiac dysfunction without bacterial dissemination into the heart. Our current findings indicate that OMVs are key mediators of this cardiomyocyte contractile dysfunction. To further investigate the effects of OMVs on cardiac function *in vivo*, we intravenously administered purified OMVs (10 mg/kg) into C57BL/6J mice and monitored body weight, survival, cardiac electrical activity, and heart function. This OMV dose was selected to parallel the cardiac dysfunction observed with a sublethal dose of LPS (10 mg/kg), known to cause severe cardiac impairment in mice (48). OMV administration resulted in marked mortality in mice (Fig. 7A). At 24 h post-OMV injection, OMV-treated mice exhibited pronounced arrhythmias characterized by irregular RR intervals and a reduced heart rate (Fig. 7B through D). Echocardiographic analysis further revealed significant cardiac abnormalities. Representative echocardiographic images (Fig. 7E) demonstrated increased LV wall thickness following OMV infusion. Quantitative assessment confirmed that OMV exposure significantly decreased stroke volume and cardiac output (Fig. 7F and G). Consistent with impaired LV function, the thickness of both anterior and posterior LV walls during diastole and systole was markedly reduced (Fig. 7H through K), while LV chamber volumes during both phases were substantially reduced in OMV-treated mice (Fig. 7L and M). Collectively, these data suggest that OMVs released during *P.a.* infection can enter the circulation, reach the heart, and contribute directly to cardiac structural remodeling and functional decline.

## DISCUSSION

Pneumonia is a leading cause of morbidity and mortality worldwide (49, 50). Bacterial infections, such as *Streptococcus pneumoniae*, and viral infections, such as influenza or SARS-CoV-2, are the most common causes of pneumonia (8, 10, 51, 52). *P. aeruginosa* is less frequently associated with CAP, where *Streptococcus pneumoniae* and *Legionella pneumophila* predominate (53). However, *P. aeruginosa* infections are prevalent in immunocompromised individuals, including those with chronic obstructive pulmonary disease (COPD) and cystic fibrosis of hospital-acquired infections (54–56). Regardless of the infecting agent, the coexistence of pneumonia and cardiovascular complications markedly increases among patients admitted to ICUs (8, 10, 57, 58).

Intranasal infections with bacterial species *Francisella novicida*, *S. pneumoniae*, and *Staphylococcus aureus* have been shown to induce cardiac dysfunction through bacterial translocation into heart myocardium (59–61). In contrast, infection with *P.a.* exhibits a distinct pathogenic mechanism. We previously demonstrated that intranasal instillation of *P.a.* causes high mortality and significant LV dysfunction, despite minimal bacterial dissemination into the heart (11). This finding suggests that soluble mediators released from the pathogen and/or host contribute to the observed LV dysfunction during *P.a.* infection. However, the underlying mechanisms by which *P.a.* lung infection leads to cardiovascular complications and mortality remain poorly understood.

In the present study, we investigated the mechanism of pneumonia-induced cardiac dysfunction using *P.a.* as a model pathogen. We found that cell-free C-media collected from *P.a.*-infected hMDMs caused marked cardiomyocyte contractile dysfunction. Exposure of hiPSC-CMs to *P.a.* C-media led to a shortened beat period, resulting in tachycardia within 45 min of treatment. After 2 h, cardiomyocyte contraction was completely abolished. Notably, cardiomyocyte morphology and viability remained largely intact initially, with cell death observed in 45–55% of the population by 24 h post-treatment. Furthermore, FPD measurements revealed that both depolarization and repolarization phases were shortened in treated cardiomyocytes. This electrophysiology alteration likely reduces cardiac output by limiting diastolic filling and ventricular filling (62), consistent with our earlier observation (11).

The uniform beat period and normal electrical conduction waves observed in cardiomyocytes treated with C-media from uninfected hMDMs confirmed that these cells were well synchronized. In contrast, cardiomyocytes exposed to *P. aeruginosa* C-media exhibited slower and prolonged electrical conduction waves, indicative of impaired electrical coupling and syncytial dysfunction. Cardiac APs are tightly regulated by the coordinated influx and efflux of ions. The rapid depolarization of the membrane potential, driven by Na^+^ influx, initiates the upstroke of the AP. A detailed analysis of MEA recordings revealed phase 2 EADs in hiPSC-CMs exposed to *P.a.* C-media. This pattern closely resembled the Na^+^ channel inactivation delay observed in cells treated with ATX-II, a sea anemone-derived peptide toxin known to inhibit the Na^+^ channel inactivation (63, 64). Consistently, *P.a.* C-media prolonged the AP in a manner similar to that observed in ATX-II-treated cardiomyocytes (Fig. S10A through C). These findings suggest that *P.a.* C-media enhances Na^+^ channel activation and delays its inactivation during the repolarization phase, resulting in prolonged APs. The increased Na^+^ entry through the sarcolemma also promotes a secondary rise in intracellular Ca^2+^ levels, a well-established driver of arrhythmogenesis (40, 65). Indeed, hiPSC-CMs treated with *P.a.* C-media displayed elevated intracellular Ca^2+^ levels and irregular Ca^2+^ cycling intervals, indicating disrupted excitation-contraction coupling. Collectively, these results demonstrate for the first time that OMVs released from *P.a.* can modulate Na^+^ channel activity, thereby altering Na^+^ and Ca²^+^ homeostasis and predisposing cardiomyocytes to arrhythmogenic events.

In response to bacteria PAMPs, host cells produce inflammatory cytokines, such as IL-1β, IL-6, and TNF-α, which activate innate immune cells, including macrophages, monocytes, and neutrophils (66–69). Recent studies have shown that host cells can also release EVs loaded with cytokines, small RNAs, microRNAs, and other bioactive molecules that further propagate inflammatory signaling (12). In parallel, bacteria release a larger number of OMVs both within host cells and into the extracellular environment (37, 69). In this study, we observed that *P.a*.-infected macrophages produced both cytokines and EVs containing bacterial OMVs, implying that these mediators collectively contribute to cardiomyocyte contractile dysfunction. Interestingly, heat inactivation of C-media, which denatures cytokines, unexpectedly exacerbated cardiomyocyte contractile dysfunction. This finding argues against the involvement of heat-sensitive cytokines (70, 71) as the primary drivers of dysfunction. Instead, we interpret these results to indicate that heat treatment disrupts EV membrane integrity, leading to the release of their contents, which potentiate cardiomyocyte injury. This interpretation is further supported by our experiments using C-media derived from heat-killed *P.a.-*treated hMDMs. This data strongly suggests that EVs containing bacterial components, rather than cytokines alone, are key mediators of *P.a.*-induced cardiomyocyte contractile dysfunction.

To further investigate the role of PAMPs in cardiomyocyte contractile dysfunction, P.a. was incubated in culture medium either in the presence of or absence of macrophages, and the resulting C-media were applied to hiPSC-CMs. Our results showed that both media derived from P.a. alone and from P.a.-infected macrophage cultures induced comparable cardiomyocyte contractile dysfunction, as evidenced by a reduced beat period. These findings indicate that bacterial products, likely released from infected macrophages within exosomes and OMVs, are the principal mediators of cardiomyocyte contractile dysfunction.

Bacteria utilize OMVs to interact with host cells and modulate the immune system, thereby promoting colonization, transmission of virulence factors, and establishment of pathogenesis (17, 72). In this study, we isolated OMVs released by *P.a.* and demonstrated their ability to induce cardiomyocyte contractile dysfunction, as evidenced by an increased beat period and a corresponding decrease in the beat rate. Furthermore, the irregular beat period and missing depolarization spikes observed in OMV-treated cardiomyocytes indicated the development of arrhythmias. Because arrhythmia induction is closely linked to disturbances in intracellular Ca^2+^ cycling (40, 73, 74), we next examined Ca^2+^ handling in cardiomyocytes exposed to *P.a.* C-media and purified OMVs. Notably, both treatments disrupted intracellular Ca^2+^ oscillation by altering multiple parameters, including peak amplitude, peak duration, time-to-peak ratio, and decay time.

Since infected macrophages release EVs and bacteria release OMVs into the medium (16, 37), we next investigated the impact of exosomal and OMV content from *P.a.*-infected hMDMs on cardiomyocytes. A key question was whether exosomes produced by macrophages during *P.a.* infection carry molecules of bacterial origin. Our results support this hypothesis: exosomes generated by hMDMs during *P.a.* infection contained bacterial proteins, as demonstrated by LC-MS/MS analysis, which identified 31 bacterial proteins and toxins present in both OMVs and exosomes isolated from the *P.a.* C-media. This finding confirms that exosomes produced during bacterial infection can carry biomolecules of bacterial origin. To validate these proteomic findings, we isolated exosomes from BALF of *P.a.*-infected mice and the serum of ICU patients who are positive for *P.a.* infection. Western blot analysis revealed that flagellin B was present in these exosomes, corroborating our LC-MS/MS data and confirming that exosomes released during bacterial infection carry bacterial biomolecules. To determine whether the proteinaceous components of OMVs contribute to cardiomyocyte contractile dysfunction, OMVs were treated with proteinase K before exposure to hiPSC-CMs. Interestingly, degradation of OMV proteins significantly improved cardiomyocyte contractile function (Fig. S8), indicating that protein components, along with other potential biomolecules such as carbohydrates, contribute to the observed dysfunction. Further studies are needed to identify the specific carbohydrate molecules responsible for the toxic effects on cardiomyocytes.

Our *in vitro P.a.* infection model revealed that exosomes released from infected macrophages and OMVs released from *P.a.* carry virulent factors/toxins. These factors can damage organs, including the heart (75). Furthermore, we found that OMVs intravenously administered to C57BL/6 mice caused significant cardiac abnormalities (e.g., LV wall thickness, less cardiac output) and increased mortality.

In summary, we demonstrated that C-media from *P.a.*-infected hMDMs induces cardiomyocyte contractile dysfunction. Our findings define the roles of exosomes and OMVs in modulating ion channel activity and their downstream effects on cardiomyocyte function. Importantly, the mechanisms identified in our *in vitro* hiPSC-CM model were recapitulated *in vivo*, where systemic administration of bacterial OMVs caused severe cardiac abnormalities and increased mortality. These results suggest that neutralizing key bacterial antigens packaged within OMVs could represent a potential therapeutic strategy for ICU patients with *P.a.* pulmonary infections. Additionally, *P.a.* OMVs may serve as vaccine candidates: immunization of mice with OMVs formulated with aluminum phosphate adjuvant elicited protective immunity (76). However, it will be essential to ensure that OMVs delivered with adjuvant do not induce cardiac dysfunction.

## MATERIAL AND METHODS

### Bacterial growth and media

*P.a.* (strain PAO1) was grown overnight in Luria broth (LB) at 37°C and used as a starter for fresh *P.a.* culture for infection experiments. Briefly, 100 μL of overnight grown *P.a.* were inoculated with 5 mL LB and allowed to grow until log phases (0.5–0.8 OD at 600 nm). The bacteria were harvested and resuspended in RPMI or saline for *in vitro* and *in vivo* experiments, respectively.

### Infection of hMDMs and processing of conditioned media (C-media)

Overnight cultured hMDMs were infected with 1 MOI of *P.a.* (with live or heat-killed at 95°C for 45 min) for 22 h. The cell-free culture supernatants from uninfected and *P.a.*-infected plates were harvested, centrifuged at 10,000 × *g* for 10 min to remove the debris, filter sterilized using a 0.22 µm filter (now called C-media), and stored at −80°C until used for hiPSC-CMs stimulation experiments. To confirm the absence of active bacteria in C-media, a CFU assay was performed using 10 µL of C-media plated on agar plate (PIA medium, BD, MD, USA), incubated at 37°C overnight, and colonies were counted.

### Treatment of hiPSC-CMs and MEA

hiPSC-CMs were seeded in MEA plate for 7–10 days for synchronization and monolayer formation. Then, hiPSC-CMs were exposed to C-media obtained from live *P.a.* or heat-killed *P.a.* (HI) and/or left untreated (UT) in a ratio of 1:1 (vol/vol) of hiPSC-CM media and C-media. Real-time cardiomyocyte functionality data were acquired on MEA as previously described (77). In brief, cells were stabilized for a minimum of 30 min in the MEA system (Maestro Edge, Axion Biosystem, GA, USA), after which the baseline was recorded, followed by C-media treatment. The data were recorded for 5 min at 15-minute intervals for the mentioned duration using AxIS Navigator software version 2.0.4.21. The data analysis was performed using the Cardiac Analysis Tool version 3.1.8 (Axion Biosystem, GA, USA) and the AxIS Metric Plotting Tool version 2.3.1.

### Animal experiments

The C57BL/6J mice used were obtained from Jackson Laboratory and housed at the Ohio State University’s Animal Resources Facility. Healthy male and female mice at 10–12 weeks old were used in this study. Mice were kept on a 12:12h light cycle at 30–1270% humidity, with standard water and chow. To minimize the potential confounders, simple randomization was performed by including the age-matched mice and treating the mice with vehicle only. All animal handling and infections were conducted in an animal facility at Ohio State University, following OSU’s IACUC guidelines. Mice were injected intravenously with 200 µL DPBS (Mock) or OMVs (final OMV per mice = 10 mg/kg body weight in a 200 µL volume) and were observed for 36 h. The blind method was used for group allocation of the mice, and it was masked from the laboratory study team. Standard early removal criteria, such as >20% wt loss, body score, and weakness, were considered for removing the mice from the study.

### Human serum sample

The Ohio State University ICU Registry and Prospective Cohort Study (BuckICU) (IRB: 2020H0175, IBC: 2020R00000034) is a collaborative effort among investigators within the Division of Pulmonary, Critical Care and Sleep Medicine and Department of Internal Medicine. We received serum samples from patients admitted to the ICU for severe pneumonia with *P.a.* infections and other complications (non-infection-related) and isolated the exosomes using ExoQuick solution, following the manufacturer’s instructions. The exosome lysates were subjected to western blot analysis to detect the bacterial flagellin.

### Statistical analysis

Statistical analyses were conducted using GraphPad Prism 10.4.1 (Prism Inc., San Diego, CA, USA). Two groups were compared using a two-tailed unpaired Student’s *t*-test. Results are presented as mean ± SD, with statistical significance defined at *P* < 0.05.

## SUPPLEMENTAL MATERIAL

Supplemental material mbio.03091-25-s0001.pdf Additional experimental methods and supplemental figures.

ASM does not own the copyrights to Supplemental Material that may be linked to, or accessed through, an article. The authors have granted ASM a non-exclusive, world-wide license to publish the Supplemental Material files. Please contact the corresponding author directly for reuse.
