# Supplementary material for: Pseudomonas aeruginosa-mediated cardiac dysfunction is driven by extracellular vesicles released during infection
Source: mBio. 2026 Jan 15;17(2):e03091-25. doi: 10.1128/mbio.03091-25 (PMC12893005; doi:10.1128/mbio.03091-25)
Supplement: Supplemental material — Additional experimental methods and supplemental figures. [file mbio.03091-25-s0001.pdf]

Supplemental information:

Isolation of Human monocyte-derived macrophages (hMDMs)

Human monocyte-derived macrophages (hMDMs) were prepared from healthy human volunteers using an approved OSU IRB protocol as described[75]. Briefly, PBMCs were isolated from heparinized blood on a Ficoll cushion and then cultured in Teflon wells (Savilexx, Minnetonka, MN, USA) for 5 days in 20% autologous serum. hMDMs in the cultured PBMCs were adhered to tissue culture plates for 2-3h at 37°C and 5% CO_2 in 10% autologous serum. Lymphocytes were washed away, and hMDM monolayers were repleted with RPMI containing 10% autologous serum and incubated overnight before being used for infection experiments.

The culture of human induced pluripotent stem cell-derived cardiomyocytes

hiPSC-CMs were plated according to the supplier's protocol as described earlier[75]. Briefly, hiPSC-CMs were cultured in 0.1% gelatin coated tissue culture plate using plating media (FUJIFILM Cellular Dynamics, Inc. WI, USA) and incubated for 48 h in a humidified chamber at 37 °C and 5% CO_2. Subsequently, we replaced the plating media with CDI maintenance medium (FUJIFILM Cellular Dynamics, Inc. WI, USA) and changed every other day for 5-7 days. hiPSC-CMs from were harvested and re-plated ($3.0X10^4$ cells per well) in a 24-well CytoView MEA plate (Axion BioSystem, GA, USA) coated with fibronectin (50 µg/mL) and incubated at 37°C with 5% CO_2 in a humidified atmosphere,

and the maintenance medium was changed every alternate day and used for our *in vitro* assays.

Cytotoxicity assay

Cell death was assessed by using the Cytotoxicity Detection Kit (Roche, Germany) following the manufacturer's instructions. Briefly, 25 µL of culture medium was transferred to a 96-well plate, mixed with 25 µL of the LDH cytotoxicity assay reagent, incubated for 30 minutes at 37°C, and the optical density was measured at 492 nm with Spectrophotometer (Molecular Devices).

ELISA

Cell-free culture supernatants (C-media) from P.a. infected or OMV exposed hMDMs were collected at 24h and used to measure IL-1β, IL-6, TNF, and IL-10 by ELISA (Duo set ELISA kits, R&D Systems) according to the manufacturer's instructions.

Isolation and quantification of outer membrane vesicles (OMVs) and Extracellular vesicles (EVs)

P.a. (PAO1 strain) was cultured overnight in 10 mL LB. The next day, 500ml LB culture was inoculated with 10mL *P.a.* culture and further grown in a bacterial incubator at 37°C for 20-22h. Then, the bacterial culture was centrifuged at 5,000g for 15 min at 4°C. The supernatant was collected and further centrifuged at 10,000g for 30 min to remove cell debris. The supernatant was collected and passed through a 0.45 µm PES filter to remove the bacteria. Then the OMVs were pelleted by ultra-centrifugation at 150,000g for 2 hours at 4°C and the OMV pellet was washed with PBS. Finally, the OMVs were resuspended

in PBS and stored at -80°C for further analysis and experiments. The same protocol was used for the isolation of EVs (mixture of exosomes and OMVs) from the C-media harvested from *P.a.* infected hMDMs. NanoFCM (NanoFCM Co., Ltd, UK) was used for the quantification of OMVs and EVs.

Separation of OMVs from EVs for LCMS analysis and western blotting

Extracellular vesicles were harvested from C-media from *P.a.* infected hMDMs and were incubated with CD9 magnetic beads to separate OMVs from EV fraction. For this, 100 µl of human CD9 magnetic beads (Invitrogen) were taken into 1.5ml tubes and washed three times with 1 ml PBS on a magnetic stand. Then, 100 µl of concentrated EVs were mixed with CD9 magnetic beads in a 1:1 ratio and incubated overnight at 4°C. The tubes were placed in a magnetic stand, and the unbound sample was collected (flowthrough), mixed again with fresh CD9 magnetic beads, and incubated overnight at 4°C. The exosome-free vesicles (OMVs) and the CD9-bound exosomes were lysed with TN-1 lysis buffer[77]. Protein quantification was done using the BCA method and lysates (25 µg) were subjected to LC-MS/MS analysis. Protein matched lysates from CD9 bound EVs and free OMVs were subjected to Western blot analysis to confirm the purity of EVs and OMV separation. We probed the membrane with an anti-CD9 antibody followed by a specific secondary antibody and development by use of ECL (Amersham Biosciences/ GE Healthcare). Similar methods were used to determine the flagellin B levels in the exosomes harvested from BALF of *P.a.* infected mice and human serum samples from ICU patients positive for *P.aeruginosa* infections

Intracellular Ca^{2+} measurements

For intracellular Ca^{2+}measurements, hiPSC-CMs were plated onto fibronectin-coated 29 mm glass-bottom dishes. The Ca^{2+} transients in the hiPSC-CMs were visualized using Fluo-3AM (ThermoFisher Scientific) according to the manufacturer's protocol and published protocol[76] with some modifications. Briefly, hiSPC-CMs were incubated with DMEM containing 10μM Fluo-3AM dye for 40 mins at 37°C in a humidified 5% CO_2 incubator. Subsequently, the medium was replaced with fresh medium and C-media in a 1:1 ratio, and the cells were further incubated for 30 minutes before imaging. Intracellular Ca^{2+} was concurrently monitored through line scan imaging using a Nikon A1R laser-scanning confocal microscope equipped with a 60× 1.4 NA oil-immersion objective under 488 nm excitation, with emitted light collected in the 500–530 nm range. All experiments were performed at room temperature with triplicate wells.

Electrocardiography (EKG) and Echocardiography (Echo)

For subsurface ECG recordings, 1.5% Isoflurane in oxygen was used as anesthesia at a flow rate of 1.0 L/min. Mice were placed supine on a heated pad to regulate body temperature, and subcutaneous electrodes were placed beneath the skin in a lead II configuration. ECGs were recorded for five minutes each on a PowerLab 4/30 (AD Instruments, Houston, TX). The mice remained unconscious during the reading. Before data analysis, the ECG tracings were manually checked for anomalies or artifacts. The ECG traces were then analyzed using AD Instruments' LabChart 9 Pro software. To assess cardiac function *in vivo*, 2D-Echo (Vevo 2100, Visualsonics) was performed in mock or OMV-injected mice, 24 hours after administration. Mice were anesthetized in an

induction chamber with 1.5% isoflurane in oxygen and a flow rate of 1.0 L/min. Mice were then placed supine on a heated stage, and hair on the chest was removed with depilatory lotion. Isoflurane 1.5% was used to maintain anesthesia. Using an MS-400 transducer, proper anatomical orientation was determined by imagining the heart's parasternal long axis. M-mode images were taken at the level of papillary muscles. The images were analyzed to determine heart functions.

Supplemental figures:

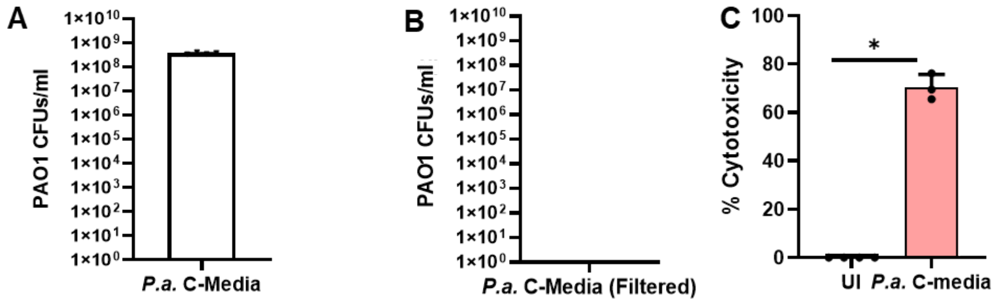

Supplemental Figure 1: *P. aeruginosa* infection causes macrophage cytotoxicity. Human monocyte-derived macrophages (hMDMs) were infected with *P. aeruginosa* PAO1 (1 MOI) for 22 hours. (A) Bacterial load in hMDM lysates was determined by CFU assay on Pseudomonas Isolation Agar (PIA). (B) Bacterial load in filtered, sterilized conditioned medium (P.a. C-media; 0.22 µm filter) from infected hMDMs. (C) Cytotoxicity of C-media from uninfected (UI) and *P. aeruginosa*–infected hMDMs was assessed by LDH release assay. Data are presented as mean ± SD; $^*p < 0.05$.

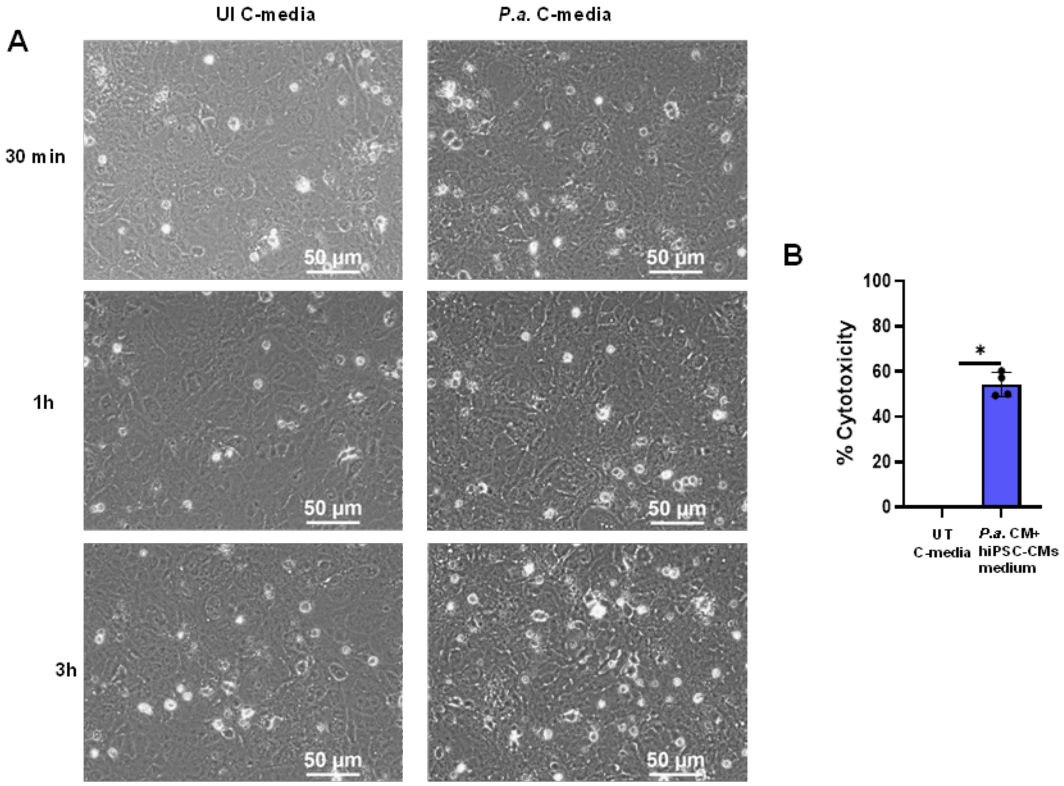

Supplemental Figure 2. Cytotoxicity of hiPSC-CMs treated with C-media from *P. aeruginosa*–infected hMDMs. hiPSC-CMs were plated in 12-well sterile plates and exposed to a 1:1 mixture of cardiomyocyte culture medium and C-media harvested from uninfected (UI C-media) or *P. aeruginosa*–infected (P.a. C-media) hMDMs. (**A**) Representative phase-contrast images of hiPSC-CMs cultured for 7–10 days prior to exposure to C-media. Images were captured at 30 min, 1 h, and 3 h post-exposure to UI C-media or *P.a.* C-media using an Olympus CKX41 phase-contrast microscope equipped with a Leica MC170 HD camera. Scale bar, 50 µm. Images are representative of three independent experiments. (**B**) Cytotoxicity of hiPSC-CMs was assessed by LDH release assay using cell-free supernatants collected 24 h post-exposure. Data are presented as mean ± SD from three independent experiments: *$p < 0.05$.

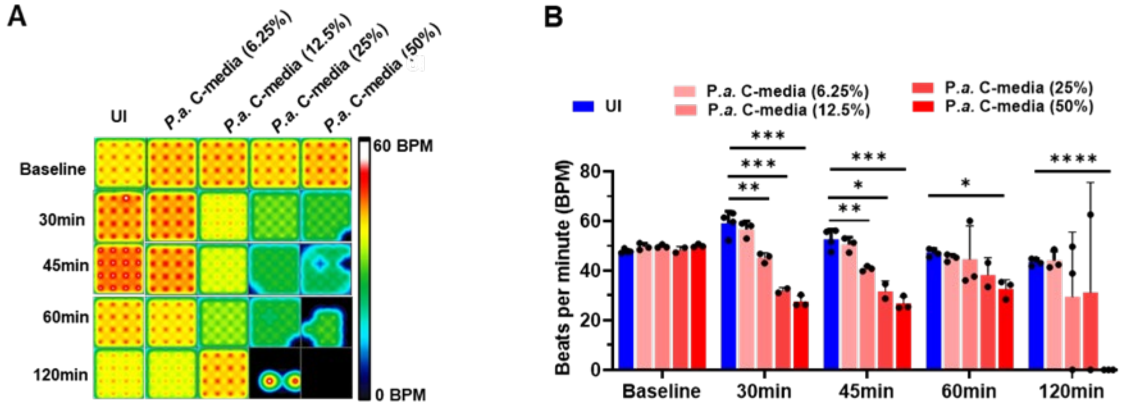

Supplemental Figure 3. Effects of *P.a.* C-media on hiPSC-CM contractility and electrical activity. hiPSC-CMs were plated in 24-well MEA plates and exposed to various concentrations of *P.a.* C-media or uninfected (UI) C-media. Cardiomyocyte contractility and electrical activity were recorded using the AxIS Navigator MEA system at 37°C and 5% CO_2 for the indicated time points. Data was analyzed using the cardiac analysis tool. (**A**) Electrical activity maps illustrate changes in beat rate of hiPSC-CMs following exposure to *P.a.* C-media or UI C-media. (B) Beat rates of hiPSC-CMs at baseline, 30-, 45-, 60-, and 120-min post-exposure to *P.a.* C-media or UI C-media. Data presented as mean ± SD; *p < 0.05, **p < 0.01, ***p < 0.001, ****p < 0.0001.

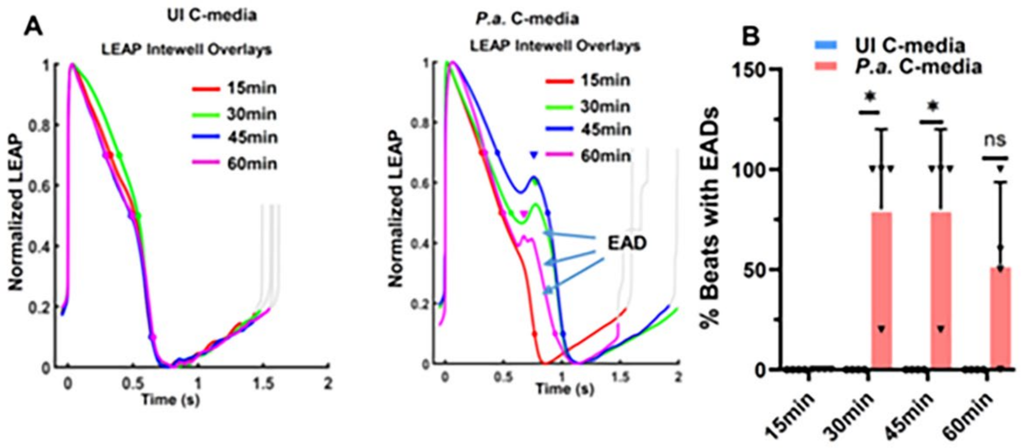

Supplemental Figure 4: *P.a.* C-media collected from hMDMs induces arrhythmia in hiPSC-CMs. hiPSC-CMs were cultured on a 24-well MEA plate (30 × 10^3 cells/well) for 7-10 days to achieve synchronization. The hiPSC-CMs were exposed to C-media from uninfected and *P.a.* infected hMDMs and recorded the physiological parameters of cardiomyocytes on MEA at 15-,30-,45-, and 60 min. (**A)** The action potential with induction of EAD shoulders at different time points. (**B)** The % beats with EAD features. Data is representative of two independent experiments, mean ± SD: * p<0.05.

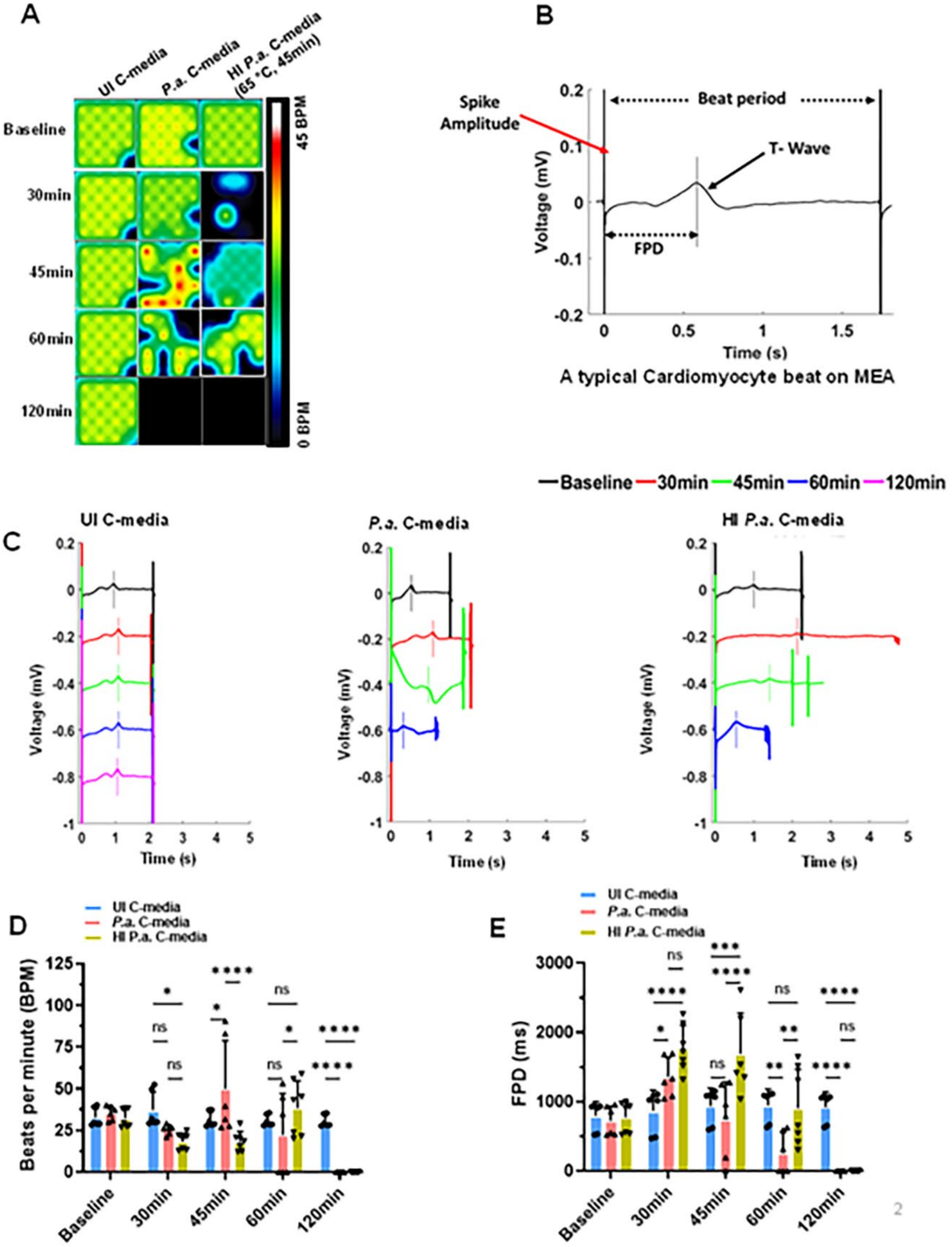

Supplemental Figure 5: Heat-inactivated C-media from *P.a.* infected hMDMs cause cardiomyocyte contractile dysfunction. hiPSC-CMs were plated in 24-well MEA

plates, and the cells were exposed to the mixture of cardiomyocyte culture medium and C-media (1:1 ratio) harvested from hMDMs that were left uninfected (UI C-media) or *P.a.* infected (*P.a.* C-media) and heat-inactivated C-media (at 65°C for 45 min). The cardiomyocyte contractility and electrical activity were recorded using AxIS Navigator on the MEA system at 5% CO_2 and 37°C for the indicated time points. Data analysis was performed using the cardiac analysis tool. (**A**) Electrical activity map showing the changes in the beat rate. Shown is a representative well from quadruplicate wells from each treatment (N=3). (**B**) A typical Cardiomyocyte beat on MEA. (**C**) Representative cardiomyocyte beat overlay recorded with the MEA system showing the beat period, T-wave, and FPD in UI C-media, *P.a.* C-media, and heat-inactivated C-media treated hiPSC-CMs. The graphs shown in (**D**) are beat rate, and (**E**) are field potential duration (FPD) at baseline, 30-, 60-, and 120 minutes post-treatment of hiPSC-CMs. Data shown in figures D and E are cumulative data from three independent experiments. mean ± SD; * $p<0.05$, ** $p<0.01$, *** $p<0.001$, **** $p<0.0001$.

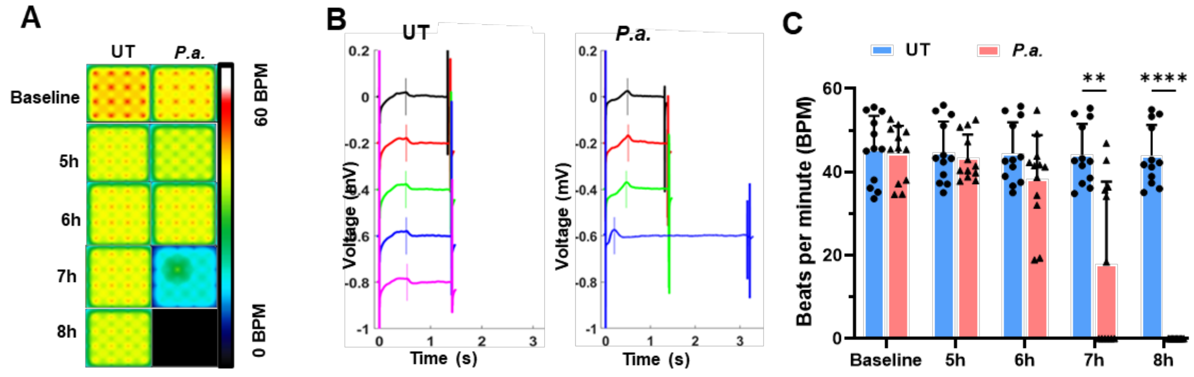

Supplemental Figure 6: Direct exposure of *P. aeruginosa* to hiPSC-CMs induces cardiomyocyte contractile dysfunction. hiPSC-CMs were plated in 24-well MEA plates and exposed to *P. aeruginosa* (1 MOI) or left uninfected (UI). Cardiomyocyte contractility and electrical activity were recorded using the AxIS Navigator MEA system at 37°C and 5% CO_2 for the indicated time points. Data was analyzed using the cardiac analysis tool. (**A**) Electrical activity maps showing changes in beat rate of cardiomyocytes infected with *P. a.* or control (UI). The activity maps shown are representative wells from quadruplicate samples for each treatment, with three independent repeats (N = 3). (**B**) Representative cardiomyocyte beat overlays recorded on the MEA system, illustrating beat period, T-wave, and field potential duration (FPD) in UI and *P. a.* infected groups. (**C**) Beat rate of hiPSC-CMs measured at baseline, 5 h, 6 h, 7 h, and 8 h post-infection with *P. a.* or UI. Data represents pooled results from three independent experiments. Values are shown as mean ± SD; $**p < 0.01$, $****p < 0.0001$.

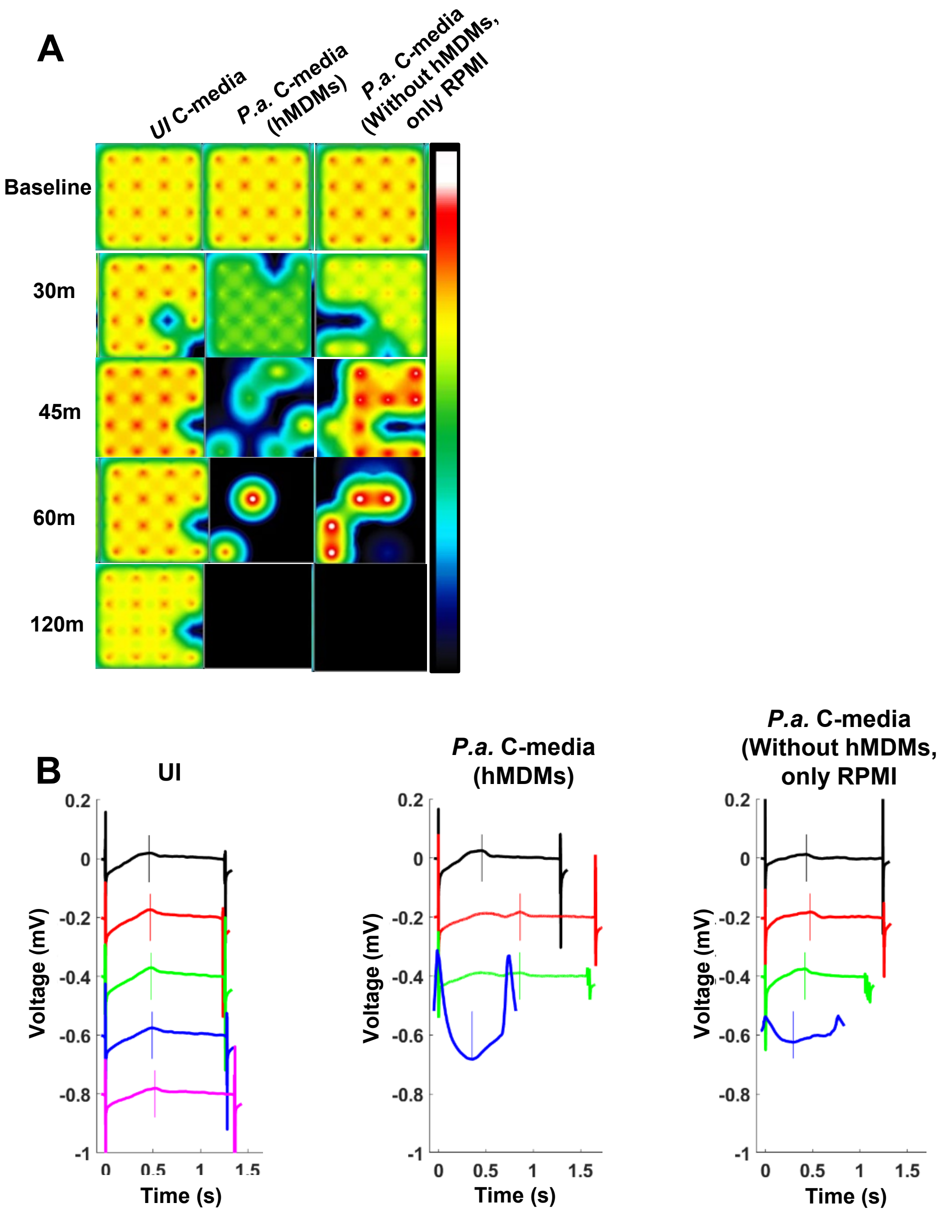

Supplemental Figure 7*:* Bacterial proteins/ toxins cause cardiomyocyte contractile dysfunction.

P.a. C-media was collected as described earlier. For culturing *P.a.* without hMDMs, *P.a.* was grown in RPMI supplemented with 10% autologous serum (Serum used for hMDM culture) for 2 days, and C-media was processed as described earlier. hiPSC-CMs were

plated in 12 well-sterile plates, and the cells were exposed to the mixture of cardiomyocyte culture medium and C-media (1:1 ratio) harvested from uninfected (UI C-media), *P.a.* infected (*P.a.* C-media) hMDMs or *P.a C-media* without hMDMs. The cardiomyocyte contractility and electrical activity were recorded using AxIS Navigator on the MEA system at 5% CO_2 and at 37°C for the indicated time points. Data analysis was performed using the cardiac analysis tool. (**A**) Electrical activity map showing the changes in the beat rate of cardiomyocytes infected with *P.a.* C-media with/without hMDMs or control (UI). The activity map shown is a representative well from quadruplicate samples for each treatment and three repeats (N=3). (**B**) A representative cardiomyocyte beat overlay was recorded with the MEA system, showing the beat period, T-wave, and FPD with *P.a.* C-media with/without hMDMs or control (UI).

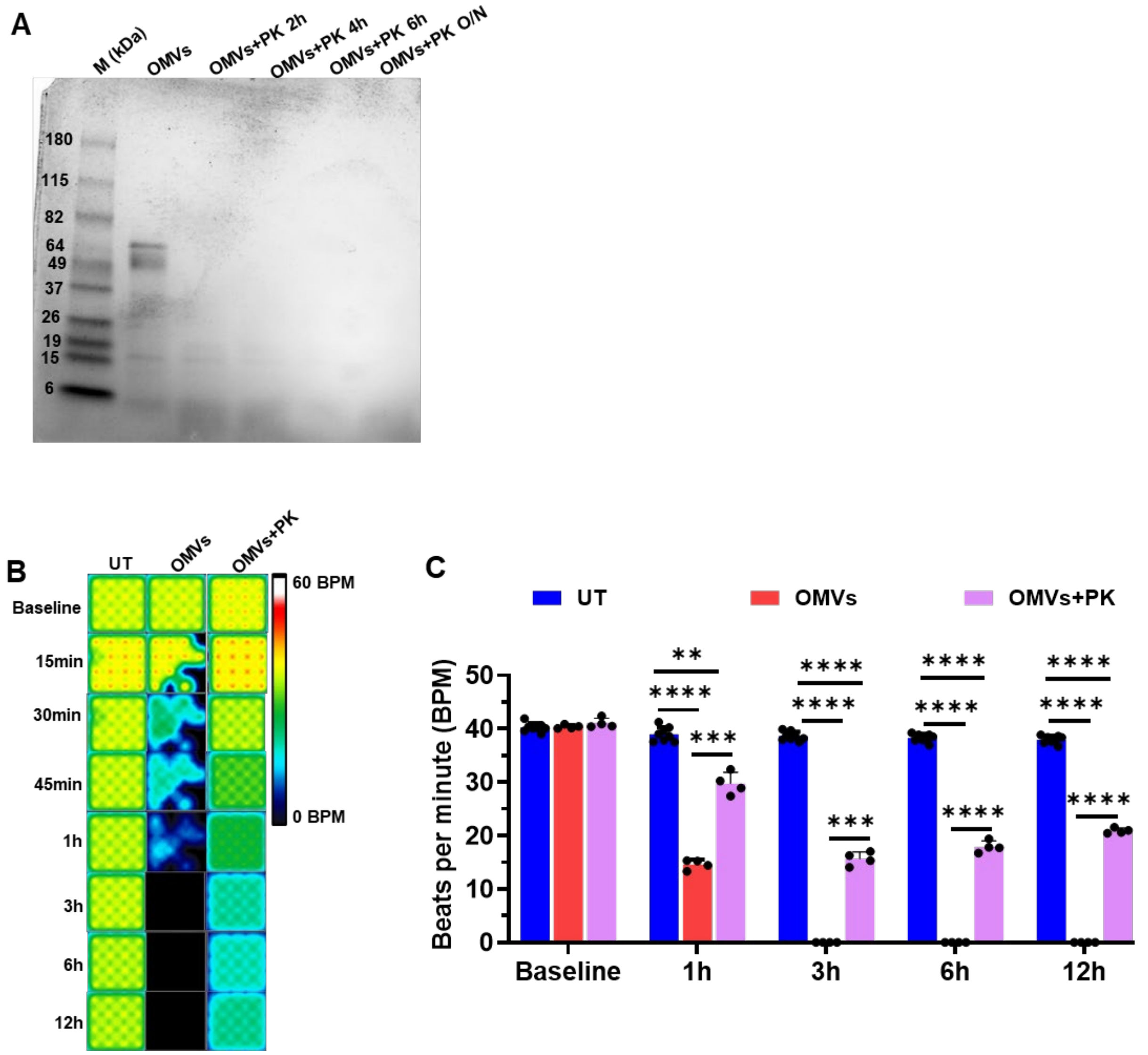

Supplemental Figure 8: Proteinaceous molecules in OMVs are partially responsible for cardiomyocyte contractile dysfunction. OMVs were digested with proteinase K (200 µg/mL) for 2 h, 4 h, 6 h, or overnight. Equal amounts of OMV lysates (10 µg) were resolved on 4–12% SDS-PAGE gels, and protein bands were visualized using Coomassie Brilliant Blue R-250 staining. (**A**) Representative image of the destained SDS-PAGE gel showing progressive digestion of OMV proteins by proteinase K. hiPSC-CMs were plated in 24-well MEA plates and exposed to either intact OMVs or

proteinase K-digested OMVs. Cardiomyocyte contractility and electrical activity were recorded using the AxIS Navigator MEA system at 37°C and 5% CO_2 for the indicated time points. Data was analyzed using the cardiac analysis tool. (**B**) Electrical activity maps showing changes in beat rate of cardiomyocytes exposed to OMVs or proteinase K-digested OMVs. (**C**) Beat rate of hiPSC-CMs at baseline, 1 h, 3 h, 6 h, and 12 h post-exposure to OMVs or proteinase K-digested OMVs. Data shown are representative of two independent experiments. Values represent mean ± SD; *p < 0.05, **p < 0.01, ***p < 0.001, ****p < 0.0001.

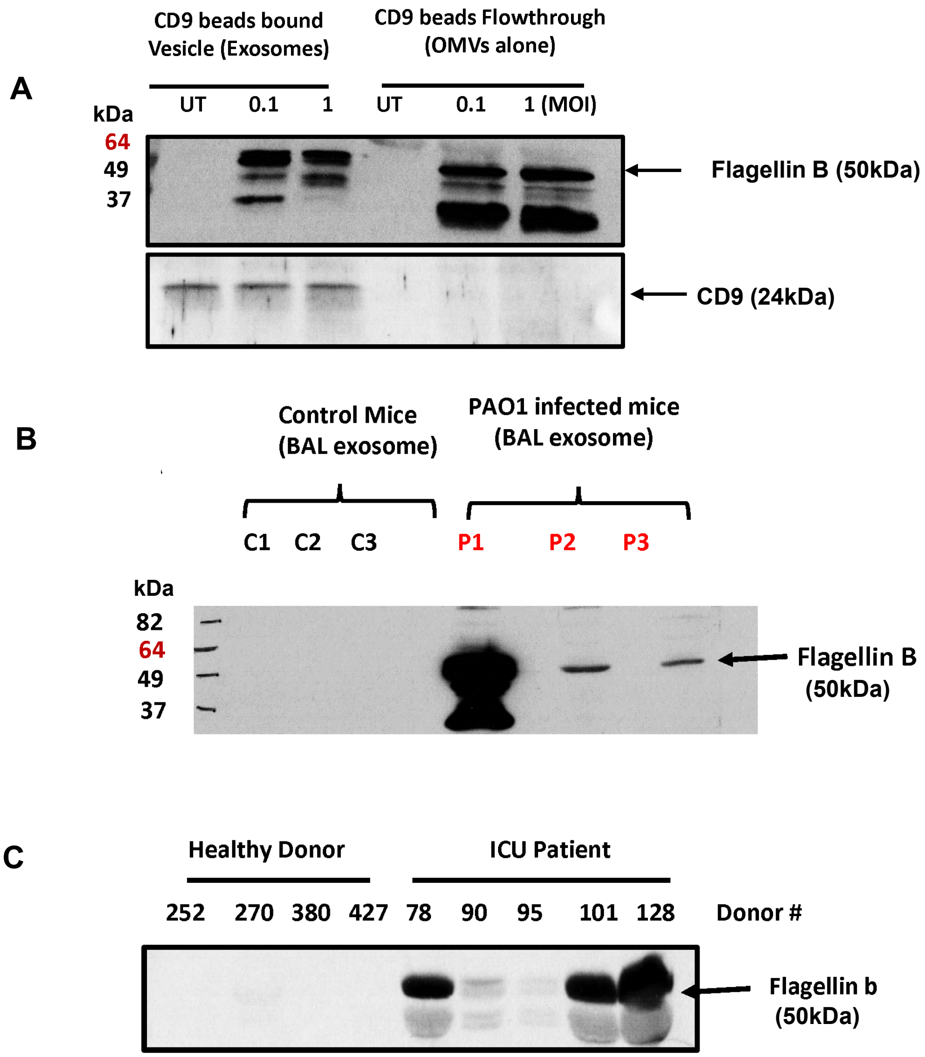

Supplemental Figure 9*: P.a.* Flagellin-B is loaded in exosomes isolated from hMDMs, mouse BALF and serum from ICU patients. Western blot analysis of exosomes and OMVs. (**A**) Western blot analysis of lysates from exosomes and OMVs separated from *P.a.* C-media using CD9 beads, (**B**) exosomes from BALF of *P.a.* infected C57BL/6J mice, and *(***C**) exosomes from human serum from healthy donors and ICU patients positive for *P.a.* infection. The membrane was probed with anti-flagellin- B antibody and CD-9 antibody.

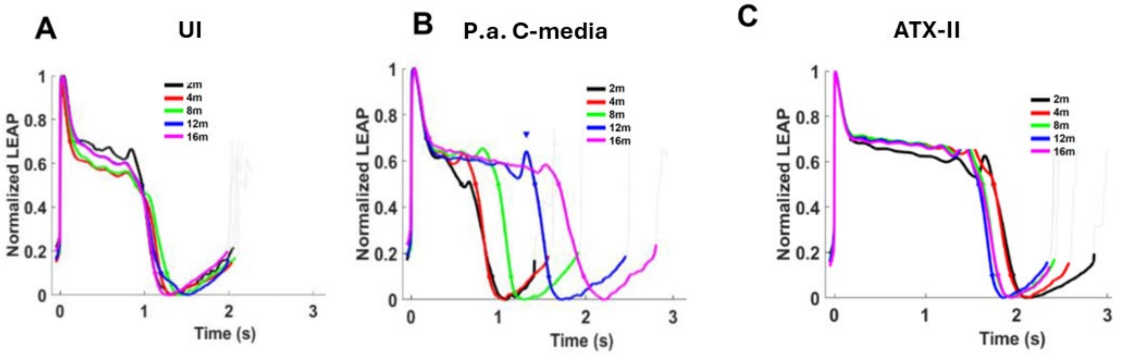

Supplemental Figure 10: ***P. aeruginosa* C-media inactivates Na^+ channels in cardiomyocytes.** hiPSC-CMs (3 × 10^4 cells/well) were cultured on 24-well MEA plates for 7–10 days to allow synchronization. The cells were then exposed to C-media collected from uninfected (UI) or *P. aeruginosa*-infected hMDMs or treated with ATX-II (10 nM; a neurotoxin known to inhibit Na^+ channel inactivation). Physiological parameters of cardiomyocytes were recorded using the MEA system at 2, 4-, 8-, 12-, and 16-minute post-treatment. (A) Action potential of hiPSC-CMs exposed to UI C-media. (B) Action potential of hiPSC-CMs exposed to *P. aeruginosa* C-media. (C) Action potential of hiPSC-CMs treated with ATX-II.
